# Chickpea (*Cicer arietinum* L.) Biology and Biotechnology: From Domestication to Biofortification and Biopharming

**DOI:** 10.3390/plants11212926

**Published:** 2022-10-30

**Authors:** Bhupendra Koul, Komal Sharma, Vrinda Sehgal, Dhananjay Yadav, Meerambika Mishra, Chellapilla Bharadwaj

**Affiliations:** 1 Department of Biotechnology, Lovely Professional University, Phagwara 144411, India,; 2 Department of Life Science, Yeungnam University, Gyeongsan 38541, Korea; 3 Department of Infectious Diseases and Pathology, University of Florida, Gainesville, FL 32611, USA; 4 Division of Genetics, Indian Agricultural Research Institute (IARI), Pusa, New Delhi 110012, India

**Keywords:** QTL, marker-assisted breeding, molecular marker, transgenic, biofortification

## Abstract

Chickpea (*Cicer arietinum* L.), the world’s second most consumed legume crop, is cultivated in more than 50 countries around the world. It is a boon for diabetics and is an excellent source of important nutrients such as vitamins A, C, E, K, B1–B3, B5, B6, B9 and minerals (Fe, Zn, Mg and Ca) which all have beneficial effects on human health. By 2050, the world population can cross 9 billion, and in order to feed the teaming millions, chickpea production should also be increased, as it is a healthy alternative to wheat flour and a boon for diabetics. Moreover, it is an important legume that is crucial for food, nutrition, and health security and the livelihood of the small-scale farmers with poor resources, in developing countries. Although marvelous improvement has been made in the development of biotic and abiotic stress-resistant varieties, still there are many lacunae, and to fulfill that, the incorporation of genomic technologies in chickpea breeding (genomics-assisted breeding, high-throughput and precise-phenotyping and implementation of novel breeding strategies) will facilitate the researchers in developing high yielding, climate resilient, water use efficient, salt-tolerant, insect/pathogen resistant varieties, acceptable to farmers, consumers, and industries. This review focuses on the origin and distribution, nutritional profile, genomic studies, and recent updates on crop improvement strategies for combating abiotic and biotic stresses in chickpea.

## 1. Introduction

### 1.1. Origin and Distribution

**Figure 1:**
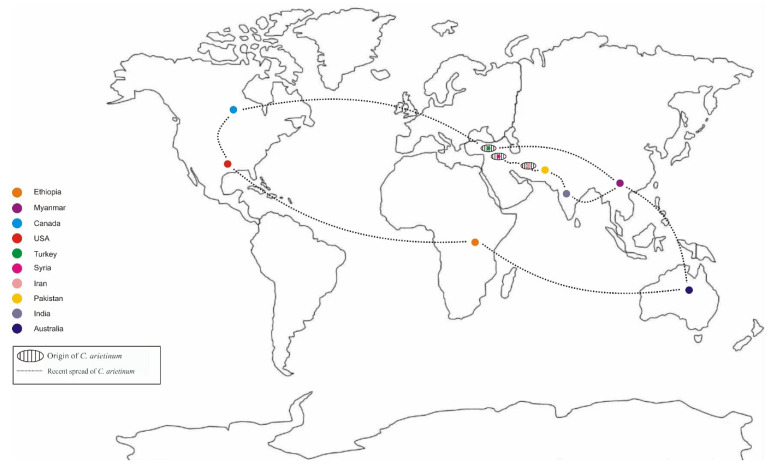
Origin of chickpea (*C*. *arietinum*) and its distribution around the world.

Chickpea (*Cicer arietinum* L.) is an essential annual pulse crop that belongs to the genus *Cicer* (Family: Leguminosae, Fabaceae) and is also recognized as “Garbanzo bean” or “Bengal gram” [1]. It is the third-largest food legume produced worldwide, after *Pisum sativum* L. (field pea) and *Phaseolus vulgaris* L. (common bean) [2,3]. It is supposed to have emerged in the Fertile Crescent’s middle area (in modern Syria, Turkey and Iran). *C. arietinum* is considered the wild progenitor of chickpea [4]. They are cultivated in sub-tropical, tropical, and temperate regions including Madagascar, the Canary Islands, Mediterranean region, north-eastern tropical Africa, and western and central Asia. They are cultivated in more than fifty countries, (0.4 per cent in Europe, 2.6 per cent in Oceania, 2.9 per cent in America, 4.3 per cent in Africa, and 89.7 per cent area in Asia). Pakistan, Turkey, Iran, Myanmar, Australia, Ethiopia, Canada, and the United States are the former top chickpea producers, accounting for 70% of global production (FAO, 2019) (Figure 1).

Since the 1990s, India has increased its production of chickpea seeds, from 4 million tonnes in 1990 to 9 million tonnes in 2019 (FAO, 2019). The rise is seen mainly due to better yields, which in 2019 touched about 10,384 t/ha universally. In India, the area harvested for chickpea cultivation accounts for 41.03% of the global area harvested. In the past 20 years, world production of chickpea has improved at an annual rate of 1.6%. Looking into the increasing population, it is expected that by 2050 the domestic requirement for chickpea will increase from 0.4 million to 0.7 million in Africa and from 7 million to 14 million in Asia [5,6].

### 1.2. Morphology

*Cicer arietinum* is the sole cultured species in the genus *Cicer* (43 species). It is a highly self-pollinated annual diploid (2n = 2x = 16) crop with a genome size of ~931 Mbps and an outcrossing rate of less than 1% [2]. Chickpea plant grows quickly, branch, and attains a height of 20 to 60 cm, or even 1 m. It contains multiple secondary lateral roots that delve into the top soil layers in addition to a large taproot that can reach a depth of two meters (15–30 cm). The leaves are five cm long and have ten to twenty sessile, oblong to elliptic leaflets. The stems are hairy, straight or twisted, simple or branching. The single, papilionaceous flowers are pink, white, blue, or purple in color [7,8].

On average, 50 to 60 pods grow. The pods are pubescent, inflated, and oblong. The seeds (usually one seed per pod) come in a range of colors, shapes, and sizes (creamy-white to black; spherical to angular; 5 to 10 mm in diameter). In India, it is usually grown in a cool season rainfed in semi-arid regions, or as a dry climate crop. It requires an optimum temperature of around 21–29 °C and 18–26 °C at night whereas 600–1000 mm of annual rainfall [5,9]. In many tropical regions, the plant is effectively cultivated in the cool season under irrigation and is well adapted to fairly temperate tropical countries. Chickpea plants are often planted in deep red or black soils (pH of 5.5–8.6) [10]. Even though some cultivars are sensitive to cold, frost, hailstones, and heavy rain, they may be able to withstand temperatures as low as −9.5 °C in the early stages or when covered in snow. For cold nights with dewfall, daily temperature fluctuations are desired and 21–41 percent of relative humidity is required for the seed to set. It is a quantitative LDP (long-day plant) that blooms in all photoperiods, despite being referred to as “day-neutral” [11,12]. It is propagated through seeds. Seeds are sown at a spacing of 25–30 cm between rows or scattered at a spacing of 10–30 cm between seeds inside rows. During the first 4–6 weeks of growth, seedlings are sensitive to weeds and should be mechanically weeded. Depending on the local climate, seeds are sown at various times of the year in various places i.e., February-March-April in the Mediterranean late; March-mid-April in Turkey, USA; September-January or April in Ethiopia; late November in India and Pakistan [5,11]. It is regularly produced in India as a catch crop and as a follow-up crop to fields of rice and sugarcane. It can be produced either as a stand-alone crop or in a rotation with other grains like linseed and sorghum [13]. Although they are commonly thought of as a dry-land crop, but they thrive on rice fields. The plant can be pulled out manually or mechanically to collect the seeds. Pods are harvested 90–120 days, 130–180 days, or when they turn yellow after sowing. The harvested plants are dried on the ground to a moisture level of 12 to 16 percent before being threshed and winnowed to remove the seeds from the chaff. The normal yield of chickpeas is 400–600 kg/ha; however, in testing, yields have exceeded 2000 kg/ha and reached 5200 kg/ha. In comparison to rain-fed fields, crop yields from irrigated fields are 20–28% higher.

There are also some studies concluding the potential influence of environment and agronomic management on various quality traits in chickpea. In a study 390 mm of rainfall showed highest seed yield whereas, a higher rainfall led to a decrease in the seed yield [14]. Similarly, seed yield is also influenced by variations in the irrigation regimes, more than 170 mm of irrigation application declined the seed yield and water use efficiency due to Ascochyta blight infection. Low availability of crop soil nutrient as well as effects of pest and diseases limits the grain yield. Santis et al. [15] studied the influence of genotype (genetic diversity) and agronomic practices on the protein content in eight different chickpea genotypes. Qualitative (especially protein content) and agronomic attributes (seed yield) under organic, conventional management were estimated. It was observed that there was no change in the protein content, but protein composition was changed. Hence, these results revealed the best performing genotype in terms of protein content and agronomic performance for cultivation under organic farming. These traits can be further explored by a proteomic method.

### 1.3. Nutritional Profile

Kabuli and desi are the two types of cultivated chickpea where 80–85% of the chickpea area is represented by desi type [1]. Desi type is usually grown in semi-arid tropics and Kabuli in the temperate region. The seed’s size, shape, and color are used to categorize it. Desi seeds are small, colored, and angular in shape, but kabuli seeds are huge, shaped like an owl’s head, and beige in color [16]. For trade, seed size and color are significant traits. Usually, desi seeds are dehulled and divided to make dhal while kabuli seeds are often cooked as a whole and for the consumption of whole seeds or as confectionery products, salads, and savory meals, consumers prefer large-seeded types. In analogy to desi, kabuli type has higher sugar and lower fiber levels and kabuli varieties have larger seeds and command higher market prices than desi varieties as price increases according to the size of the seed [16,17]. Their seeds are the chief source of amino acids, protein, fiber, calcium, iron, and phosphorus and are low in fat (4–10%) and immature seeds are eaten as snacks, roasted/boiled, or salted [18]. They include 52–70% carbohydrates and 18–22% proteins, which collectively account for around 80% of the total mass of dry seed and 4–10% fat and contain a minimum amount of lipids (>5% DM) [18,19]. It is an important crop among the pulses, which is cholesterol-free and has a high nutritional value, dietary fiber (DF), vitamins, and minerals (Table 1).

Methionine (1.3–1.6 per cent) and cysteine (2.5–3.0 per cent containing sulfur) content are usually low but when consumed with cereals that lack in lysine amino acid can fulfill the balance nutrition to the body [18]. They are also enriched with vitamins (vitamin C, B, A, K, B2, B3, B1, B9, and β-carotene precursor vitamin A, calcium, magnesium, phosphorus and potassium, cryptoxanthin, lutein, and zeaxanthin) and other unsaturated fatty acids; omega 6 (linoleic) and omega 9 (oleic acids) [19] that are required for several metabolic pathways of the body. Its straw has a higher protein level (about 5% DM) as a comparison to cereal straw; however, it is a fibrous feed (thirty to forty percent as DM crude fiber). The pod husks have a similar protein content as that of straw but have a higher fiber content [18].

### 1.4. Pharmacological Characteristics

**Figure 2:**
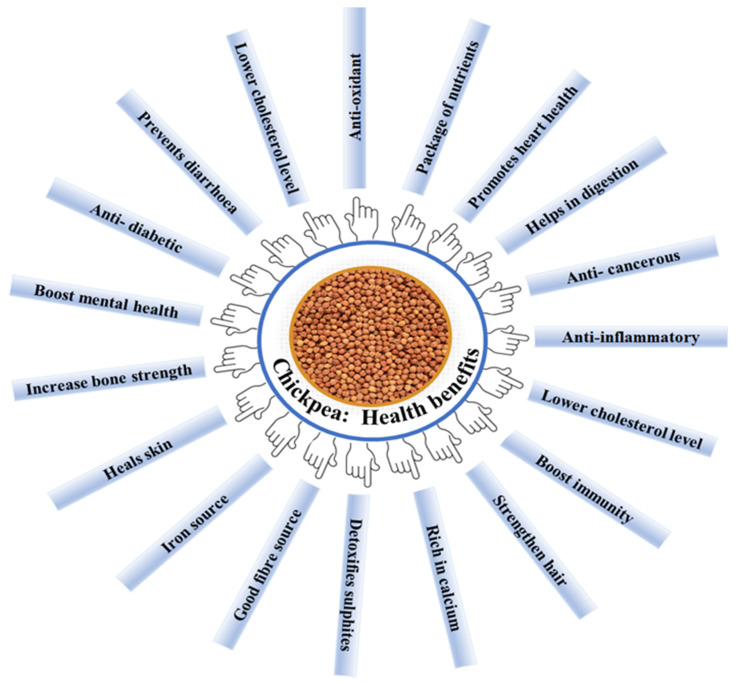
Commercialized chickpea- based snacks of different countries.

Chickpea contains several bioactive compounds that are associated with human health benefits [20]. These bioactive components (phytic acid, anthocyanins, carbohydrates, catechins, fiber, alkaloids, flavonoids, steroids, quercetin, saponins, tannins, trypsin inhibitors and terpenoids) can be taken directly from seed or its extract in form of peptides [21]. They have additional qualities that can treat abdominal pain, nausea, constipation, headache, and flatulence brought on by an excessive release of bile, in addition to conferring anti-inflammatory, antihypertensive, hypocholesterolemic, antioxidant, and anticancerous action (Figure 2), hence, considered antibilious [3,12,21].

Malic, oxalic acids are found in the glandular secretions of chickpea leaves, stems, and pods and also have some traditional therapeutic characteristics. These sour-tasting acid exudates might be used as vinegar or as medicine and these acids were traditionally harvested in India at night by draping a thin muslin cloth over the crop. The liquid was collected in bottles after the saturated towel was wrung out in the morning and this exudation from the plant’s leaves can damage clothes. These exudates can treat several diseases such as dyspepsia, cholera, sunstroke, Bronchitis, constipation, diarrhea, flatulence, snakebite, catarrh, and warts and can also reduce blood cholesterol levels. In one study on rats, they were also found to be essential in controlling blood cholesterol levels. In Chile, a newborn baby was also fed cooked chickpea mixture which effectively controlled diarrhea [12,22].

### 1.5. Genomic Analysis

Chickpea has a genome size of approx. 740 Mb in which 73.8 percent of the genome is found in scaffolds [23]. ICRISAT (International Crops Research Institute for the Semi-Arid Tropics, India) and ICARDA are two institutions that maintain chickpea germplasm (International Centre for Agricultural Research in Dryland Areas, Syria) [24,25]. A huge number of cultivated chickpea and chickpea accessions are present but the major limitation that prevents its utilization is lack of knowledge of important economic traits. Screening this huge number of germplasm lines is an expensive and tedious process, which is a major drawback. Currently, the ICRISAT gene bank houses the largest collection of chickpea germplasm in the world (20,764 accessions) from 59 countries [26] and morpho-agronomic characteristics of 98% of chickpea germplasm have been characterized to far, compared to 35% of biotic stresses and 63% of seed protein content [17,27,28]. For both quantitative and qualitative features, a large range of variations can be seen across the complete collection of accessions. All the data for evaluation and characterization can be acquiredthrough ICRISAT Genebank. Chickpea genetic resources (genetic stocks, breeding materials and mapping population) have been generated for operation in breeding and genetic studies. QTL (quantitative trait loci) maps, molecular markers and genetic linkage maps among other large-scale genomic resources have also been established and made available to breeders recently to help them successfully utilize these breeding strategies for the improvement of cultivars [29].

### 1.6. Mapping Populations

The appropriate construction of a mapping population is crucial for the generation of a genetic linkage map. The first step to developing a mapping population is the selection of two genetically different parents as it will show polymorphism but they should not be much genetically different from each other, otherwise that can lead to sterility in the progenies as well as segregation during linkage analysis [13,29]; F2, F3 progeny, backcrossing, doubled haploid, NILs (near-isogenic lines), and RILs (recombinant inbred lines). NILs are developed for QTL analysis whereas RILs are developed by following single seed descent (SSD) progression of F2 plants. This is analyzed for a further six or more generations to develop single plant offspring [30]. RILs are usually used for the study but the RIL mapping population has more results as compared to others. RIL mapping is immortal and can be replicated again over years. The traits that are targeted for mapping population study are resistance to abiotic, biotic stress, and protein coat [31,32]. New developments in the MAGIC (multi-parent advanced generation inter-cross) population development are taking place at ICRISAT. It is being generated from 28 two-way, 14 four-way and 7 eight-way crosses by utilizing eight parents. It includes cultivars as well as elite breeding lines from Africa and India. The MAGIC population has an accumulation of recombination events that have increased the novel rearrangement of alleles in it and led to the enhancement of genetic diversity. The lines produced by this are an important genetic resource for gene identification and trait mapping, and they can also be employed directly as a portable source for the development of improved cultivars [26,29].

### 1.7. Molecular Markers

As the cultivated chickpea has a constricted genetic base due to which many conventional approaches that were made to improve chickpea productivity did not get desired results [32]. Many efforts were made at the international level for generating genomic resources, and to enhance this effort ICRISAT and its partners have made attempts to accelerate the growth of genomic resources during the last few years [26]. Molecular markers with characteristics of high polymorphism and capability to high desired result analysis are required for genomic studies and crop improvement. The first molecular marker that was used for the chickpea genetic studies was isozyme but due to their small number, exhibited a low level of polymorphism in cultivated chickpea [32,33]. RFLP, RAPD was also the first molecular marker along with isozymes used for genomic resource studies. The widespread use of molecular markers for chickpea breeding and genomic studies was started after the development of SSR and microsatellite markers. SSR marker was developed from the information retrieved through genetic libraries, BAC-end sequences, tentative unique sequences (TUS), and bacterial artificial chromosome libraries (BAC), and expressed sequence tags (ESTs) [28,34,35,36] are the first choice nowadays for use as they are multiallelic and co-dominant [37]. For the molecular analysis of chickpea, around 2000 SSR molecular markers are developed and according to Varshney et al. [38] published article showed the availability of over 48,000 SSR molecular markers for PCR primer design that are perfectly suitable for usage as genetic markers.

Another genomic analysis technology i.e., (DArT) diversity arrays technology analyzes DNA polymorphism using a microarray platform. It can screen a large number of molecular markers, as it is a rapid and high throughput genome analysis method [39,40]. DArT markers (15,360) have been developed for chickpea from ninety-four different genotypes and out of which 5397 DArTs were found to show a high level of polymorphism [31]. A recent new class of markers i.e., single nucleotide polymorphism (SNP) has become the new choice for the study of genomic analysis because of the presence of distinct characteristics. High-throughput analysis, co-dominant nature, and high abundance characteristics make them different and unique from other markers [33,41]. For chickpea, several thousand SNP’s have been recognized through the transcriptomic analysis method. The whole draft genome sequence of chickpea has been developed and 76,084 SNP’s were identified in 15,526 genes. Along with the identification of SNP’s and SSR’s markers, analysis has also shown INDELs chickpea genome polymorphism markers [25,28,38].

An effective method for mapping physiologically and reasonably significant features in various genetic populations at a greater resolution than biparental mapping is Genome Wide Association Analysis (GWAS) tool [42]. GWAS was widely utilized for chickpea crop in order to assess the degree of genetic diversity, identify the genes responsible for certain traits, and identify marker-trait relationships for abiotic, biotic, nutritional, and agronomic variables. High statistical power GWAS versions: BLINK, Farm CPU facilitate the breeders to base their selection on the most important marker-trait relationships, which greatly speeds up the breeding to increase the nutritional quality of chickpeas. A reference set of two hundred eighty chickpea accessions, including advanced cultivars, breeding lines, and landraces was examined by Srungarapu et al. [43] over two seasons for grain protein, Zn, and Fe content as well as for agronomic characteristics. For GWAS analysis, 4603 highly relevant SNPs distributed within the chickpea genome were analyzed utilizing a mid-density five thousand SNP array, and 20 and 46 SNP markers were found to be strongly linked with the grain nutritional and agronomic features over the seasons. On chromosomes 1, 4, 6, and 7, respectively, there were 7 SNPs related to grain protein, 12 for Fe, and 1 for Zn content. After being validated in breeding populations, the important marker train associations (MTAs) can be utilized in the marker-assisted selection (MAS) in order to develop nutrient rich cultivar of chickpea. A similar study of the population structure of one hundred eighty-six genotypes was conducted by Ahmed et al. [44] and concluded high genetic diversity between genotype pairs, stating a varied genetic ancestry. One locus on chromosome Ca4 at 10,618,070 bp was found to be associated with salinity tolerance under hydroponic and field environment by multi-trait GWAS whereas, on chromosome Ca2 at 30,537,619 bp, they also discovered another region unique to the hydroponic system. According to gene annotation study, rs5825813 is located inside the EMB8 (embryogenesis-associated protein), while rs5825939 is found within the RPLP0 (ribosomal protein large P0). Hence, these markers can be utilized by the researchers to incorporate new genes in commercial cultivars.

### 1.8. Genome Mapping

When compared to wild and cultivated species, newly acquired molecular markers revealed minimal levels of polymorphism within cultivated species. Because of this, interspecific mapping populations have been used in all initial work on genome mapping in chickpea. In order to create the first linkage map, 26 isozymes, 3 morphological trait loci, and F2 populations were used [27,45]. Simon and Muehlbauer later joined molecular markers such as RAPDs and PFLPs to this map. The first mapping population of RILs that included 351 markers covered a distance of 2077.9 cM was developed from the interspecific cross between *C*. *reticulatum* (PI 489777) × *C*. *arietinum* (ICC 4958) and considered as the reference mapping population for further genome mapping in chickpea [46,47]. Later many studies were done by taking this map as a reference. Another map developed by Nayak et al. [37] had 521 markers and covered 2602.01 cM. Thudi et al. [31] and Bharadwaj et al. [28] constructed a map that included 1291 markers and covered 845.56 cM; whereas, Hiremath et al. [36] constructed a genetic map covering 1328 marker loci by taking it as a reference population. Numerous attempts have been made to create a map utilizing intraspecific mapping population, but the results have not been satisfactory due to the low amount of polymorphism in cultivated chickpea. Intraspecific mapping populations produced maps with fewer markers and lesser complete genome coverage [48,49]. This was the main disadvantage of using intraspecific mapping populations for generating the genetic map. So, to overcome this disadvantage genetic maps have been developed by utilizing both interspecific and intraspecific populations. Millan et al. [50] developed a genetic map based on 5 intraspecific and 5 interspecific populations (*C*. *reticulatum* × *C*. *arietinum*) along with the integration of 555 marker loci. Additionally, utilizing BAC and binary bacterial artificial chromosome (BIBAC) libraries, a physical map of chickpea has been produced [34,51,52]. There are 1945 contigs covering around 1088 Mb in this physical map. Varshney et al. [26] developed a complete map of variation in 3171 cultivated and 195 wild accessions of chickpea to provide publicly available resources for genomic studies. The genetic diversity of chickpea cultivars and wild accessions has also been discussed.

### 1.9. QTL Analysis

To identify and define root-specific genes that varied between “ICC 4958” and “Annigeri,” ICRISAT generated over 3000 chickpea ESTs from a library made using SSH (subtractive suppressive hybridization) of root tissues from these genotypes [28,29,53]. This database provides a significant new method for data mining related to root characteristics and drought tolerance for chickpea genomics researchers. Several molecular markers for the gene and QTLs have been developed connected to resistance to disease. The results of some of the QTLs formed in response to biotic stress are summarized in Table 2.

### 1.10. Marker-Assisted Breeding (MAB)

Markers linked with *fusarium* wilt resistance genes, QTLs for vernalization response, and QTLs for drought tolerance have been developed in chickpea [40,64,65]. In marker-assisted selection (MAS), markers associated with GOI (gene of interest) or QTLs are employed to track gene or QTL introgression [29]. Marker-assisted selection is more precise than the conventional method of breeding. A pyramiding of resistance genes from many sources, quality attributes, root traits for drought tolerance, and combo-resistance for two or more biotic or abiotic stresses are some qualities that are difficult to analyze phenotypically [40]. MAS is also used to track resistance gene introgression from transgenics to cultivars and top breeding lines and is crucial for improving drought tolerance in chickpeas by employing the “QTL hotspot” gene8, increasing genetic diversity by using (MAGIC) multiparent advanced generation intercrossing lines, and introgressing resistance to wilt and *Ascochyta* blight diseases, among other things [30].

## 2. Abiotic and Biotic Constraints to Chickpea Production

Biotic and abiotic pressures are major obstructions in chickpea production. Globally, abiotic stresses cause annual chickpea yield losses, either individually or in combination, which culminates in severe financial penalties [66]. Nearly 90% of the world’s chickpeas are produced in rainfed environments, where the plants are subjected to terminal drought stress and grown in soil whose water content is quickly diminishing. Due to a lack of water supply, the average chickpea grain yield is low in the primary producing countries [67]. During flowering, chickpea (rabi crop) is prone to heat stress (30–35 °C) and in India delayed harvesting of crop causes more heat stress, particularly during grain filling resulting in the reduction of crop yield. Additionally, due to increased crop intensity, the growing area for late-sown chickpea in northern and central India is growing. Drought and heat stresses can decrease the yield of the crop by up to 70% [6,68]. The low temperature in West Asia and North Africa impairs yield by causing freezing injury or death, as well as delaying podding. Hence, after drought and cold stress, heat and salinity problems are relatively important abiotic stresses. The crop is also vulnerable to biotic stresses, which further reduce the yield and include collar rot, dry root rot, *Ascochyta* blight, *Helicoverpa*, *Fusarium* wilt, *Botrytis* grey mold, and seasonal weeds. The main fungi that damage chickpea plants are *Ascochyta rabiei*; causes *Ascochyta blight* and *Fusarium oxysporum*; which causes wilting, which is the most dangerous disease (producing 100% deaths in some cases) [11,69,70]. Blight is characterized by brown blotches on stems, seeds, pods, and leaves. Additional harmful fungi causing diseases are *Botrytis cinera*- gray mould; *Alternaria sp*., *Ascochyta pisi*- leaf spot; *Leviellula Taurica*, *P*. *ultimum*-damping off; *Sclerotinia sclerotiorum*-Sclerotina rot; *Uromyces ciceris-arientini*- rust; *Rhizoctonia bataticola*, *R. solani*- dry root rot, *Sclerotium rolfsii* – root rot and *Verticillium albo-atrum*- wilt [5,11]. Some of these fungi might be significant economically as well. Viruses that are isolated from chickpea are *bean yellow mosaic, alfalfa mosaic*, *pea leaf roll*, *pea enation mosaic*, *pea streak*, and *cucumber mosaic* [71]. Some other important threat to chickpea plant is lesser armyworms (*Spodoptera exigua*) and leaf minor, Adzuki bean seed beetle (*C. chinensis*), Cutworms (*Agrotis sp*.), groundnut aphid (*Aphis craccivora*), pea aphid (*Acyrthsosiphon pisum*), and cowpea bean seed beetle (*Callosobruchus maculatus*) [71,72]. Aphids (*Aphis craccivora*) are small sap-sucking insects belonging to the superfamily of *Aphidoidea* and commonly known as greenfly and blackflies. They are another important pest of chickpea found in South East Asia, Ethiopia, and an occasional pest of the USA (*Black aphids*, *Aphis craccivora*). To control these, insecticides are used but they have evolved resistance to them and are no more effective in killing aphids. *Bruchid* sp., a storage insect is also a serious pest of stored chickpea. Normal behavior is for adult beetles to lay their eggs on the seeds, and the young larvae to eat the seeds and harm the seeds. After the development of the larva, it comes out of the seed and lays more eggs, this whole cycle goes on repeatedly causing damage to the quality of the seed of chickpea [73]. Cowpea bruchids also attack cowpea, field pea, and soybean. Cowpea bruchids continue to breed in stored pulses at grain temperatures over 20 °C. It has a life cycle of 28 days at 30 °C. To control the bruchids, the most commonly used practice by the farmers is the use of insecticides during the reproductive phase as well as several chemicals are fumigated or dusted on the seeds. Such chemicals are phosphine and methyl bromide [5]. Seed viability is also decreased by *Callosobruchus chinensis* [74]. However, this excessive use of pesticides disrupts the natural balance and has negative, often irreversible, consequences for the ecosystem and human health. Biotic and abiotic pressures are major constraints on the productivity of chickpeas. Individual pests, diseases, and weeds are predicted to cause yield losses of 50–100 percent in tropical regions and 5–10 percent in temperate zones [66,75]. As a result, the market price of this pulse crop has skyrocketed, making it difficult for a big portion of India’s rural people to cope. Malnutrition in underdeveloped nations is rising at an alarming rate due to the shortage of important pulses, which causes protein and other essential nutritional deficits in the poor and marginal population. Increasing concern about the environmental impact of the use of pesticides, combined with the demand for a sustainable farming approach and the development of chickpea cultivars with more seed output and long-term tolerance to abiotic and biotic stresses has generated global interest in improving the trait of chickpea.

### Crop Improvement through Transformation Regime

Abiotic (drought and salinity) and biotic (*Helicoverpa*, *Aphids*, *Calloso bruchus*) are major constraints affecting chickpea productivity [75]. Due to the chickpea’s sexually incompatible gene pool, the potential for genetic development through marker-assisted breeding and selection techniques is restricted. Many strategies have been developed till date to develop chickpea cultivars tolerant to both abiotic and biotic stresses (Table 3).

Various genes have been identified from different species that can be utilized for the transformation regime such as several insecticidal proteins encoded by genes in *Bacillus thuringiensis* during sporulation (*Vips*) vegetative development and (*Cry* or *Cyt*). Chickpeas were successfully genetically converted utilizing the *cry1Ac* gene for the pod borer *Helicoverpa*, the *bean-amylase* inhibitor gene for bruchids, and the *ASAL* gene for aphids [77]. (*H. armigera*) pod borer is one of the most widespread and most affecting pests of chickpea worldwide. It is a leading field pest and causes yield loss of up to 40% [90]. It is a polyphagous pest that feeds on leaves, develops seeds, and causes considerable damage to a variety of plant types [75]. Insecticides are widely used to manage pests in India and China, but their impact on the environment and the emergence of resistance in pest populations are unavoidable. *HaNPV* (*Helicoverpa nuclear polyhedrosis virus*) has also been used to control the pest but several factors i.e., lack of an efficient mechanism for product quality control and high production cost makes it unaffordable to the farmers in comparison to synthetic insecticides [90,91]. Hence, transgenic chickpea was developed using *Cry1Ac* gene. To develop transgenic chickpea resistant to these pests, *cry1Ac* gene along with *Vip3A*, *cry2Aa*, *cry2Ab*, *cry2Aa1*, *cry1Ab*, *cry1F*, *cry1AbMod, cry1AcMod* from *B. thuringiensis*, and promoter gene *atsA1*(*Arabidospis thaliana* Rubisco small unit gene) from *Arabidospis thaliana* was expressed by the biolistic transformation method [5]. They were also transformed with the *cry1Ac* gene using *Agrobacterium tumefaciens*, resulting in a large number of transgenic chickpea lines. *Cry1Ac* reduced larval growth, *Cry1Ac* caused 100 percent death of new-born larvae, *Cry2Aa* caused 98 percent demise of neonate larvae, and *Cry1Ab*/*Cry1Ac* caused 96–100 percent death of *H*. *armigera* pod borer larvae [5,90]. These new forms of the genes c*ry3Bb*1 or *bMod* /*cry1Ac* may help lower *H. armigera* and delay the selection of resistant pests in chickpea, even if the synthesis of these hybrid or novel toxins in chickpea has not yet been attempted [5]. The interpretation of fusion or hybrid proteins has various advantages (wider range of toxicity, new specificities and greater pest toxicity in developed transgenic cultivars). As a result, for successful pest management, pyramiding two or more genes with different modes of action is preferred. Another approach applied in transforming chickpea impervious to *H. armigera* was the use of a combination of *cry1Ac* gene (*Bacillus thuringiensis*) + *npt*II + *CaMV35S* as a promoter through *A. tumefaciens* gene mediated transformation method using cotyledon nodes as explants [75]. The result showed that *cry1Ac* protein above 10mg^−1^ leads to 80–85% protection from pests with a high mortality rate of >80% [75]. Transgenic chickpeas were also developed using *Bt* in co-ordinance with entomopathogenic fungus 3 (*Metarhizium anisopliae*) that showed resistance to *H. armigera*. Both susceptible and *cry2A-*resistant *H. armigera* larvae were killed by *M. anisopliae*, which was used in conjunction with *Bt* chickpea [92]. Transgenic chickpea also developed resistance to aphids. *ASAL* (*Allium sativum agglutinin lectin*) from plant *Allium sativum* was used to develop transgenic chickpea mediated by *A. tumefaciens* that shows very low aphid resistance i.e., 11–42% [5,77] whereas the low toxicity of lectins and biosafety issues related to mammalian toxicity were the major drawbacks of using them in this study. Similarly, transgenic chickpea was developed using *αAII* gene mediated by *A. tumefaciens* to control the bruchids by utilizing the most effective gene α amylase inhibitor gene (*αAII*) from another legume common bean (*Phaseolus vulgaris*) [73]. In the insect intestine, the *αAII* gene limits the function of an enzyme that digests amylase starch, causing bruchids to grow slowly and eventually die [5,73].

The primary restrictions on chickpea output worldwide are abiotic stresses (terminal drought and heat stress), and they are anticipated to get worse because of climate unpredictability and change [66]. The efficiency of breeding programs has been improved via concerted efforts to create cultivars that are quickly resistant to abiotic stress. It is also very sensitive to salt, and the overproduction of proline makes it more tolerant of abiotic stresses. Hence, to increase the tolerance of chickpea to salinity, Vigna *P5CS* cDNA under the control of the *CaMV35S* promoter was transferred to the chickpea cultivar using *A. tumefaciens* as a transformation method [83]. *P5CS* from Mothbean (*Vigna aconitifolia*) is a bifunctional enzyme that usually catalyzes the first 2 phases of proline biosynthesis, which was first isolated using *E. coli* mutants by a functional complementation technique. The result showed the overproduction of proline and alleviation in tolerance to salt stress in transgenic chickpea plants. A genetic engineering method has provided hope for improving its resistance to water deprivation. A transgenic chickpea plant was developed utilizing osmoregulatory gene *P5CSF129A* from *Vigna aconitifolia* plant with the use of axillary meristem explants through *A. tumefaciens*-mediated for the overproduction of proline [82]. The proline concentration in the developed transgenic plant was increased, and the plant was able to overcome the unfavorable effects of drought stress. To increase the transpiration efficiency under drought stress, transgenic chickpea was developed by *DREB1A* gene + *rd29A* promoter obtained from *Arabidopsis thaliana* plant through *A. tumefaciens*-mediated method with the use of axillary meristem explants. The result showed an increase in transpiration, stomatal response, and water uptake [93]. miRNAs (microRNAs) are a type of short non-coding RNAs that are gradually identified at the post-transcriptional level as key regulators of gene expression, and overexpression of miR408 was done using a mature embryo as an explant to improve drought tolerance. MiR408 is found practically in all terrestrial plants. Transgenic lines that overexpress miR408 were developed in order to research the impact of miR408 in drought stress in chickpea. Increased miR408 expression was linked to induced tolerance [86].

## 3. Biofortification

Chickpea seeds are deficient in methionine (Met) and cysteine (Cys), two main essential sulfur-containing amino acids and omega-3 content (ALA) [19,94]. Amino acids, of which 20 are categorized as essential and the remaining as non-essential, are the building blocks of protein synthesis. There are nine amino acids that the human body cannot produce. So, to fulfil the body’s requirement for these amino acids, they are to be derived from a diet that contains cereals, legumes, and animal proteins. Protein-calorie malnutrition predominates in emerging countries like India where vegetarian diets are preferred, as plant proteins can only provide fifty to seventy percent of the important amino acids to the body which is not sufficient [95]. In humans, methionine insufficiency leads to neurological problems, fatty liver, and cancer; in animals, it can cause low milk output, decreased meat quality, and poor wool production in sheep [96]. There have been efforts to increase methionine content since there are 13 biosynthetic routes through which methionine can be changed to cysteine in plants. The pea vicilin gene promoter, which was mediated by *A*. *tumefaciens*, controlled the expression of the sunflower seed albumin (*SSA*) gene from *B*. *napus* to raise the methionine content in chickpea [5]. The result showed the elevation of methionine content in normal soil conditions whereas an increase of both methionine and cysteine in high nitrogen to low sulfur state [89].

### 3.1. Foliar Method

Biofortification is defined as a process for the production of micronutrient-enriched staple foods [97]. Nearly one-third of all cultivated soils are deficient in Zn, which is a worldwide problem. In the soil where chickpeas are grown, Zn shortage is fairly common. Zn deficiency is common in most of the countries where chickpea is grown i.e., India (48.5 percent), Pakistan (70 percent), and Turkey (80 percent) [98,99]. Zinc deficiency is the most prevalent micronutrient illness preventing legume production because of its role in vital physiological processes such as protein synthesis, photosynthesis, enzyme activation, and pollen function. It is one of the key causes of human malnutrition in countries such as India where dietary beans represent a significant source of nutrient intake. To alleviate Zn shortage, inorganic Zn fertilizer has been applied to the soil, seeds, and leaves. ZnSO_4_.7H_2_O was used to apply pre-optimized quantities of Zn as seed coating (5 mg Zn kg^–1^ seed) (33 percent Zn) and seed priming (0.001 M Zn). Control treatment includes non-primed dry seeds and hydropriming (soaking in water) [100]. It was found to be one of the most successful ways for increasing production, Zn biofortification, and grain quality in both desi and kabuli chickpeas. To further enhance the Zn concentration in chickpea plants, foliar application of Zn was used in which 0.1% ZnSO4 foliar spray as a source was sprayed on the plant [94]. It increased the boldness and vigor of seeds as well as the seed zinc content in both zinc-deficient and sufficient seeds. In another study, foliar application with a combination of Zn + urea (0.5% ZnSO4 + 2% urea) was employed to improve the biofortification of chickpea with both Zn and Fe and that resulted in the development of seeds with higher Zn and Fe content [101]. The foliar application involves giving nutrients directly to plants, although it is difficult to evenly spray all of the plant’s green portions without risking toxicity. In contrast, the priming and coating procedure for seeds does not result in any yield loss. Hence, the seed coating and priming method has recently emerged as an inexpensive and the best approach for biofortification (Table 4).

### 3.2. Microbial Treatment

Zn applied to the plant through fertilizers is not sufficient to counteract the Zn deficiency as 96–99% of applied fertilizer gets converted into an unavailable pool through ppt or complexation with carbonates, phytates, and oxides [106]. In this condition, plant growth-promoting bacteria (PGPB) is used along with the nutrient application (Table 5).

Through the release of organic acids, microbes solubilize the nutrients, acidification, and chelation in the rhizosphere and through carboxylation to increase the intake of Zn and other nutrients to promote plant growth. PGPB (*Enterobacter sp*. *MN17*) improves the uptake of nutrients, enzyme synthesis, phytohormone production, nitrogen fixation, and siderophores to increase plant growth [85,98]. Other than Zn, boron is also an essential micronutrient required for plant development that controls the metabolism of carbohydrates and nitrogen, cell division, fruiting, and flowering. In addition, it also acts as a catalyst for many chemical reactions. Boron deficiency is the main constraint and is improved by seed coating and by microbial treatment. Different level of boron (0.0 control;1.0,1.5,2.0,2.5, and 3.0 g B/kg) with BTB (boron-tolerant bacteria) i.e., *Bacillus sp.* MN54 was used for the seed coating and found effective to cope with extreme environmental conditions [91,111]. The outcome showed that seed coating with boron and BTB inoculation enhanced nodulation, growth, and yield while also raising the boron content in the seeds.

Similarly, Fe with PGPR (plant growth-promoting rhizobacteria) is used for iron biofortification. Iron deficiency is responsible for affecting millions of individuals around the world as a major nutritional disorder [106]. In the human body, Fe acts as a co-factor for enzymes to carry out several body reactions and its insufficiency in the body can cause anemia, disability, and stunted mental growth. PGPR (*Bacillus cereus* UW 85, *Azotobacter vinelandii* MAC 259, *Pseudomonas*, *Bacillus megaterium*, *E. coli*) were used for the biofortification of chickpea with Fe content. These PGPR improved plant growth by adding various favorable effects such as improved nitrogen fixation, phosphate solubilization, phytohormone synthesis, organic acid production, and reduction in susceptibility to diseases. This combined use of PGPR and Fe led to 81% increase in grain and 75% increase in shoot iron content [106]. As a result, the biofortification of plants using PGPR is thought to be a safe method for increasing the iron content of various edible parts of plants. Another approach that was used to improve the chickpea plant was the use of the combination of *Rhizobium* sps. *BHURC01* + PGPR + *Pseudomonas fluorescens*. Here, rhizobacteria inhibited the phytopathogenic fungi and that leads to the suppression of plant disease. The combined effect showed the promotion of nodule formation leading to plant growth in chickpea [107].

## 4. Constraints in the Development of Transgenic Chickpea

The advancement of transgenic chickpea by *Agrobacterium*-mediated transformation has many constraints because of its highly calcitrant nature. For the efficiency of transformation and recovery of stable transgenics several factors have been optimized i.e., type of explant (somatic embryo, cotyledonary node, cotyledons, hypocotyl leaves, stem, etc.,), size of explant, the orientation of explant, pre-incubation of explant, media supplementation (CCC, Ag salt), PGR composition, duration of culturing, agro-inoculation/co-cultivation/sonication duration, the temperature of the co-cultivation medium, vacuum treatment, developmental stage and antibiotic sensitivity of explants [10,90]. Another major constraint is the release of the polyphenolics during explant preparation and pre and post-incubation have been resolved by pre-treatment of the excised explants with inhibitors of polyphenol oxidase (DTT, sodium thiosulphate and L-cysteine) before co-cultivation [112]. It has increased the transformation efficiency by decreasing the accumulation of polyphenols. Still, there are many constraints which in the production of transgenic chickpea such as choice and response of explants to tissue culture, varied and variable response of chickpea varieties to tissue culture regimes (all varieties are not amenable to invite regeneration), unavailability of robust tissue culture protocol, slow response of the explant to media and media components and supplements (PGRs, CCC), the poor establishment of in vitro raised plantlet/or its roots in soil or soil-rite mixture [70,75,112]. Despite all the efforts of the researchers, no genetically modified chickpea variety has been authorized for widespread commercial cultivation. However, a field trial of transgenic chickpea was approved by the government of India in 2015.

## 5. Industrial Application of Chickpea

**Figure 3:**
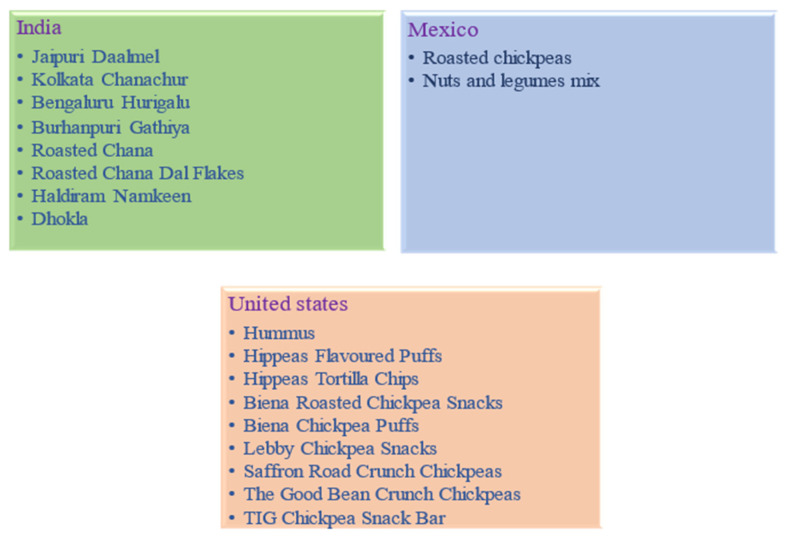
Value-added products of chickpea.

In the United States and Europe, chickpea seeds in cans are widely used and are mostly processed into besan flour (a reliable alternative to wheat) and used to make bhajis, pakoras, and bread in the Indian subcontinent [3]. This flour can also be utilized for making gluten-free cakes and biscuits made for children suffering from celiac disease [7]. Dhal is a meal cooked from split chickpeas that have had their seed coverings removed and seeds are frequently dried and then cooked for snacks and sweetmeats to make a thick soup [102]. Green pods and young plants are consumed in the same way as spinach and seeds that have been sprouted are consumed as a vegetable or added to salads and as sprouting is reported to enhance the proportionate levels of biotin, pantothenic acid, niacin, vitamin K, Fe, ascorbic acid, choline, tocopherol, pyridoxine, inositol, and of the seed [3]. Additionally, its seeds are offered as a side dish made with lemon, salt, and pepper. A common traditional food in the Middle East, Turkey, and North Africa is hummus, a dip or spread made from boiling and mashed chickpea seeds (combined with tahini (sesame seed paste), olive oil, lemon juice, garlic, and salt). Its roasted roots are also used as a substitute for coffee. The researchers’ and industries are constantly working on the production of chickpea snacks. Various challenges have been faced during chickpea processing because of the several processes that harm the nutritional composition of the chickpea. Several chickpea snack products have been made by the food industry as seen in Figure 3.

This food industry process of transforming chickpea flour into products has also led to several patents [7]. In many developing countries, chickpea has also been used in the form of animal feed. An adhesive may also be developed which is suitable for plywood, though not water resistant. Gram husks, green or dried stems, and leaves are utilized for stock feed; entire seeds can be processed straight for feed [12,102]. It is also said that leaves yield indigo-like dye and chickpea harvest 21 percent starch appropriate for textile sizing, giving a light finish to cotton cloth, wool and silk [49].

## 6. Characterization of Chickpea Varieties

**Figure 4:**
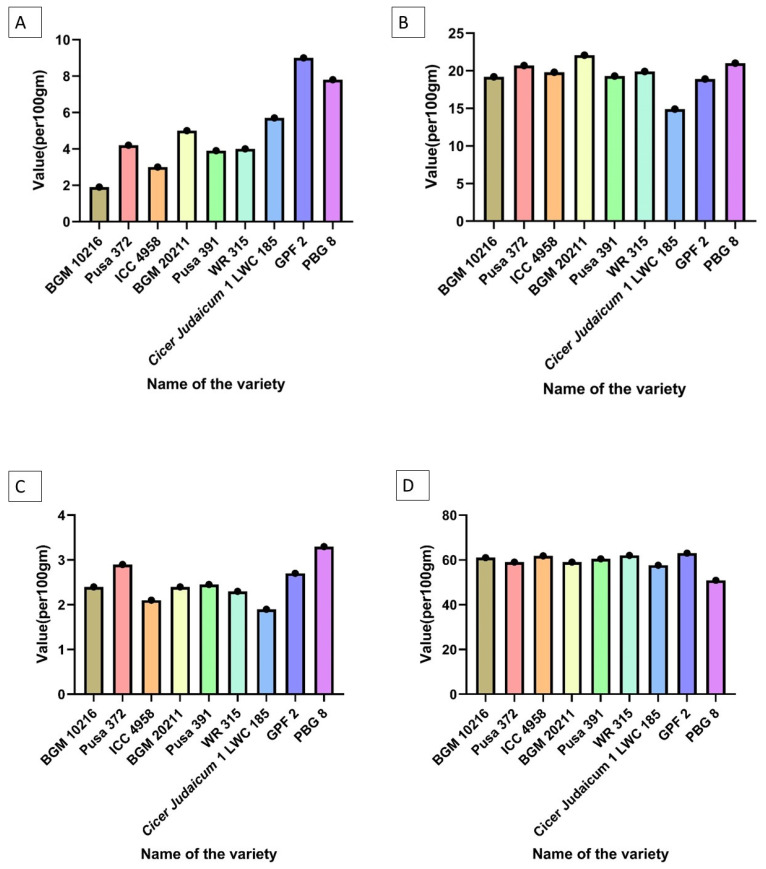

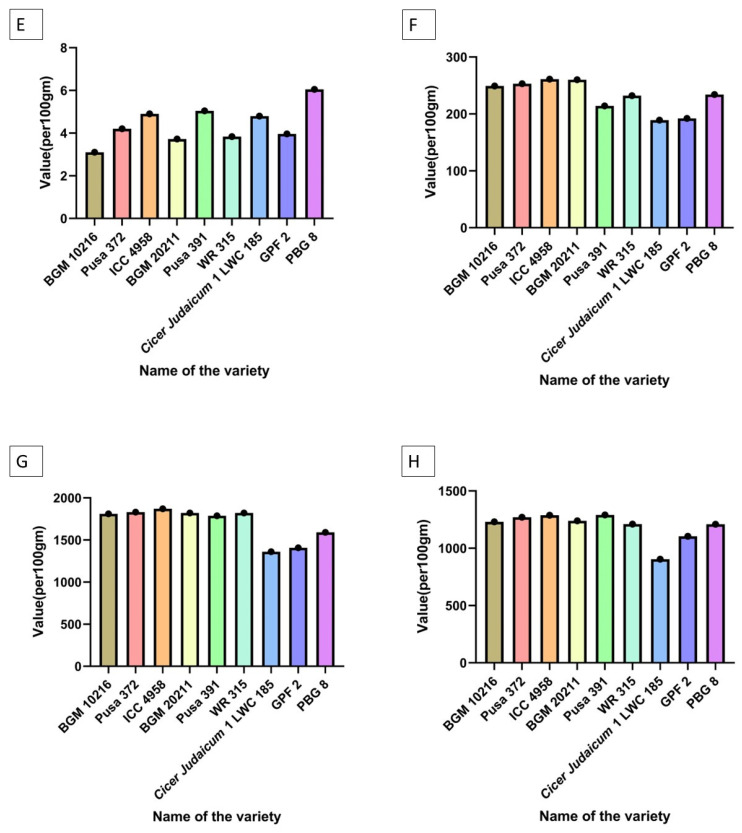

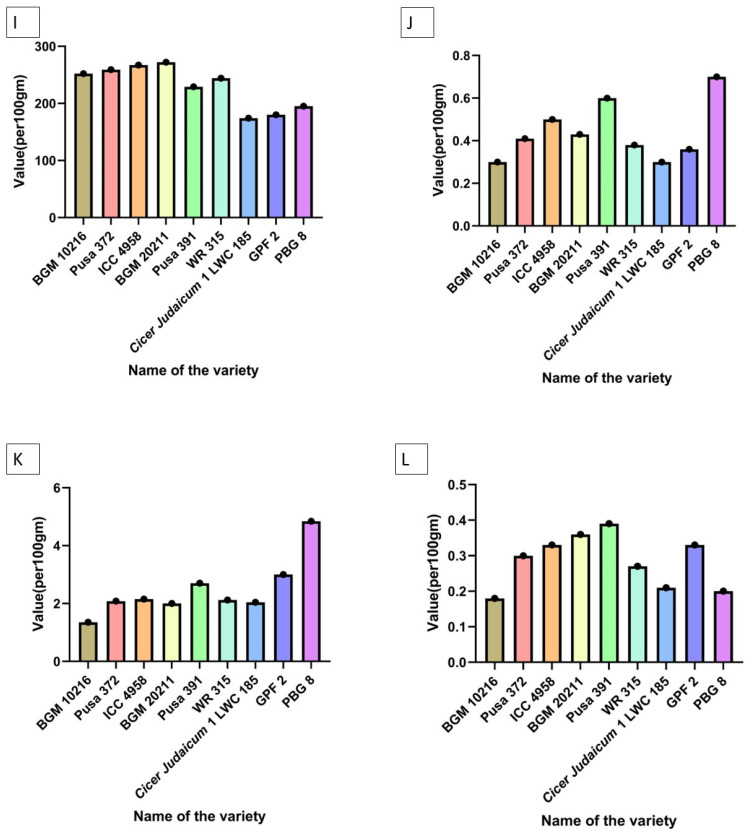

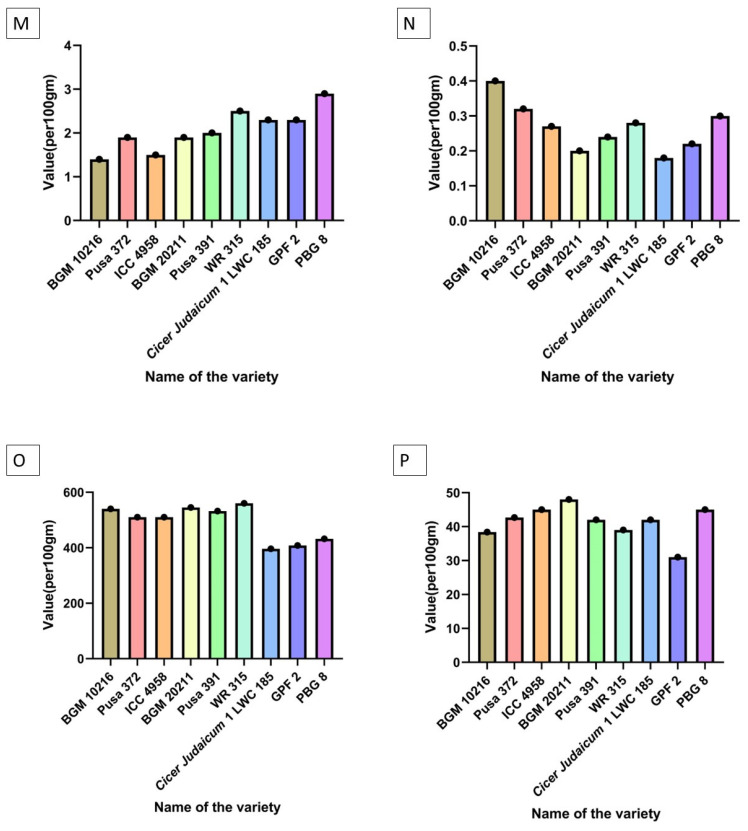

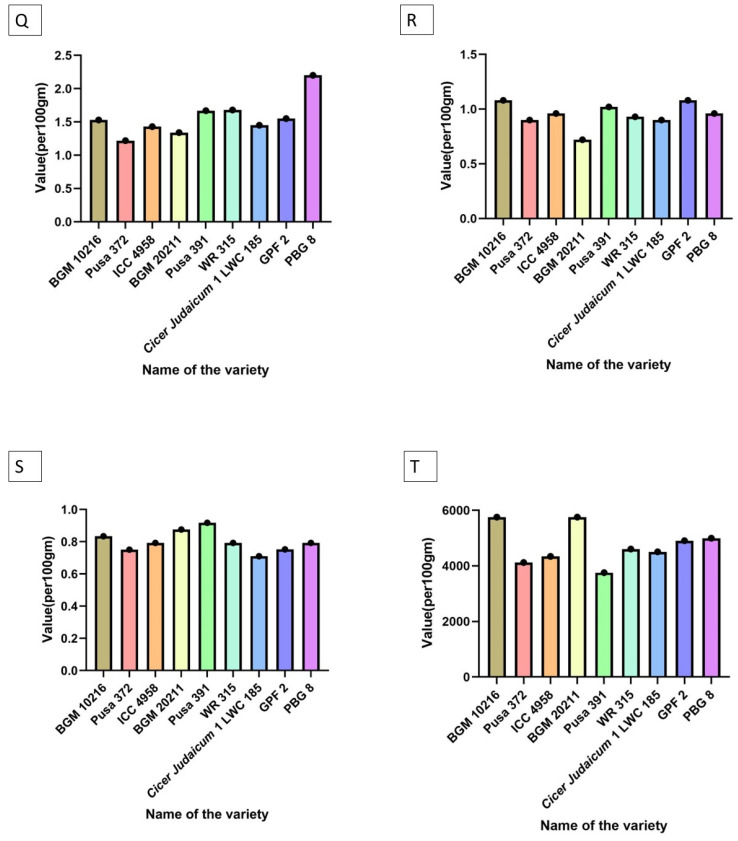

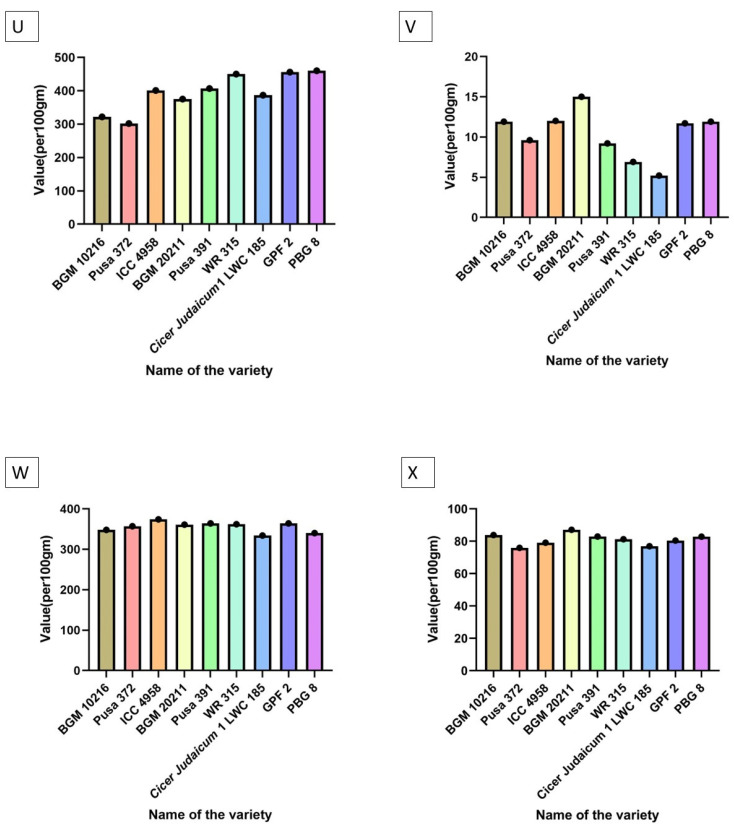

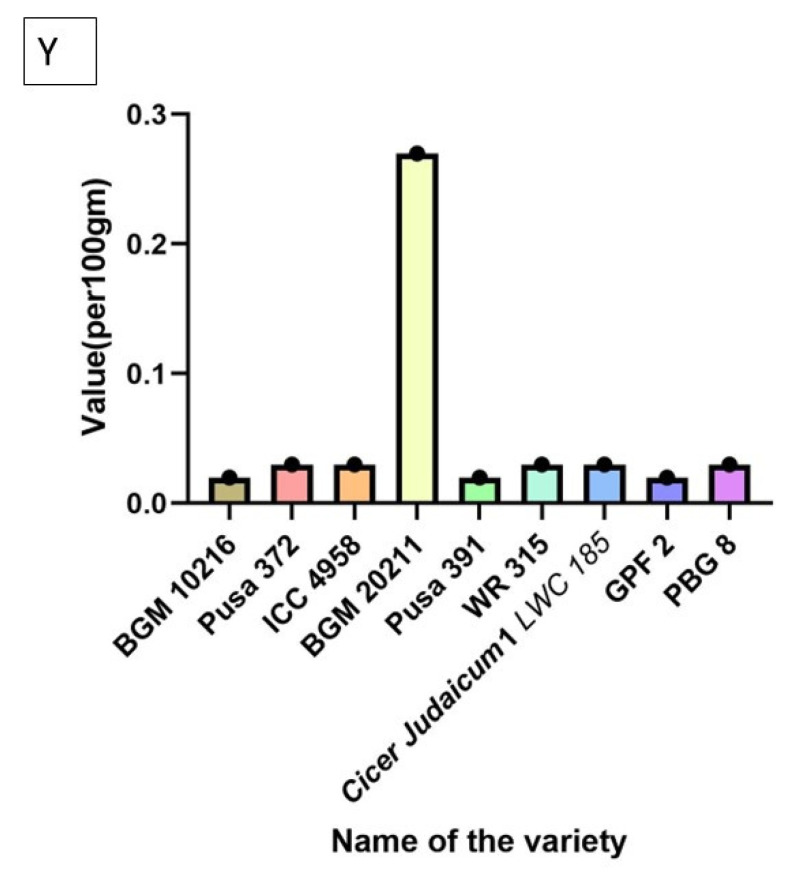
Biochemical tests of nine chickpea varieties (**A**) Crude Fiber content, (**B**) Crude protein content, (**C**) Total ash content (**D**) Carbohydrate content (**E**) Crude Lipid—Fat content (**F**) Methionine content (**G**) Arginine content (**H**) Lysine content (**I**) Cysteine content (**J**) Saturated fatty acids content (**K**) Polyunsaturated fatty acids content (**L**) Riboflavin content (**M**) Niacin content (**N**) Thiamin content (**O**) Folate content (**P**) β—carotene content (**Q**) Phenolic content (**R**) Flavonoid content (**S**) Omega 6: Omega 3 content (**T**) Antioxidant activity content (**U**) Lectin and hemagglutination activity (**V**) Uric acid content (**W**) Energy value content (**X**) Protein bioavailability (**Y**) Phytic acid content.

At ICRISAT, chickpea germplasm collection contains a total (>3500 accessions) whereas ICAR-NBPGR has (1500 accession), 2200 accession of whole genome sequenced, and 292 vastly diverse sets of collection and 223 superior halotypes [30]. New varieties are being developed every year to deal with the biotic and abiotic constraints of chickpea. As these constraints lead to the reduction of chickpea production by up to 90%. ICAR has released chickpea varieties Pusa 1105, and 2024 that are high-yielding and well-adaptable kabuli varieties while Pusa 362, 1103, 372, and BGD are well-adapted desi types. As per the annual report of IARI, several novel varieties have been developed in the year 2020 for biotic and abiotic constraints. Our research group has worked on two very newly developed and elite varieties of Chickpea (*C. arietinum* L.) i.e., Pusa Chickpea 20211 (aka *Pusa Chickpea Manav*) and PUSA 10216 that we have procured from IARI. The latter is a drought-tolerant variety that had been developed after the introgression of “QTL-hotspot” for drought tolerance in the ICC 4958 genotype of chickpea in the genetic background of Pusa—372 with the assistance of molecular markers. “Pusa Chickpea—10216” is moderately immune to fusarium wilt, stunt diseases, and dry root rot of chickpea with a mean grain yield of 1447 kg/ha under the moisture stress condition of the Central Zone of India. Its average weight is around 22.2 g per 100 seeds and matures in 110 days. Similarly, Pusa Chickpea Manav is also developed by the introgression of the “QTL-hotspot” from WR 315 into Pusa 391 for fusarium wilt resistance. It is high-yielding and disease-resistant (moderately resistant to pod borer, stunt, dry root rot and collar rot and highly resistant to fusarium wilt). Under wilt-stress conditions, it has a production potential of 3915 kg/ha and reaches maturity very quickly, in just 108 days. Its average weight is around 19.5 g per 100 seeds and with a protein content of 18.92 per cent. We have worked on the biochemical characterization of these two novel varieties of chickpea BGM 10216 and BGM 20211 and their respective parents i.e., Pusa 372, ICC 4958, WR 315 and Pusa 391. Several tests (crude fiber, crude protein, methionine, cysteine, riboflavin, β-carotene (vitamin A), omega 6: omega 3, uric acid, and protein bioavailability) were run on them to assess their nutritional profile. As a result of the comparative study (Figure 4), it can be concluded that both of these novel varieties have some advances over their parents but BGM 20211 has shown better results between these two newly developed ones.

Furthermore, the trend for all the tested parameters on varieties can be observed in Table 6.

## 7. Conclusions and Future Prospects

Chickpea production has thus far involved a lot of work. Due to the growing population and the fact that it is a strong source of nutrients and have a high nutritional value, the chickpea world trade has grown significantly over the past two decades. Additionally, the production of chickpeas has expanded in previously untapped areas such as Australia and North America, and this trend is predicted to continue in the near future. It is a significant crop of pulses that is majorly impacted by biotic and abiotic stress. However, the productivity of this economically valuable crop can be increased through new biotechnological approaches. The synergy of modern biotechnological techniques along with conventional techniques to overcome the various stress can bring a new green revolution in sustainable chickpea production. The development of a larger number of genetic mapping, molecular markers, and markers linked with the characteristics, as well as transcriptomics resources, has allowed genomic technology to get integrated for chickpea improvement. The chickpea’s entire genome was sequenced in 2013, marking a significant accomplishment in the field’s genomics and inspiring more studies for developing genomic resources that can be used to advance the crop. Advancements in the development of several thousand markers including SNPs, DArTs, and SSRs have been done, and based on these genomic resources’ QTL, physical maps, as well as dense genetic maps have also been developed for crop improvement in chickpea. Along with these, other modern approaches such as MABC and MARS breeding methods are currently used and utilization of this modern genomic technology has not only helped in the development of superior chickpea cultivars whereas, but it has also shortened the time for developing new cultivars. Previously the purpose of the CRISPR/Cas9 technique for genome manipulation in chickpea was not well-adapted by the researchers. The first research utilizing Cas9 technology for drought resistance was published in 2021 and involved the chickpea plant. Two genes, 4CL and RVE7, were the targets. The revolutionary method of Cas9-mediated gene knockout will make it easier for plant breeders to create chickpea cultivars resistant to drought (non-transgenic approach, ethically acceptable). Chickpea has got a sufficient amount of omega6 content in them but lacks omega3 content and is very far away from the ideal range of omega6: omega3 ratio. As per our study on different varieties of chickpea, we have seen a disruptive ratio. Therefore, the biofortification of chickpea with omega-3 fatty acids will be a novel approach. This will not only improve the amount of omega3 fatty acid content in chickpea, but also correct the ideal range of polyunsaturated fatty acid ratio required for good health. This is an unexplored area that should be addressed in the future.

## Figures and Tables

**Table 1:** Nutrition profile of chickpea (*Cicer arietinum* L.).

Nutrients	Nutrient Value Per 100 g
Calories	378–396
Protein (g)	18.77–24
Fat (g)	4.1–6.04
Carbohydrate (g)	39.56–54.2
Fiber (g)	7.4–12.22
Ash (g)	3.4
Minerals	
Ca (mg)	57–160
P (mg)	250–310
Fe (mg)	4.0–12.3
Na (mg)	24
K (mg)	700–718
Zn (mg)	2.76–4.1
Mg (mg)	79–138
Vitamins	
*β*-carotene (μg)	67
Thiamine (mg)	0.45–0.5
Riboflavin (mg)	0.2–0.26
Niacin (mg)	1.54–2
Tocopherol (mg)	11.2–12.9
Folic acid (mg)	206–290
Pantothenic acid (mg)	1–2
Pyridoxine (mg)	0.3–0.38
Amino acids	
Lysine (g)	6.6–7.2
Methionine (g)	1.2–1.4
Cysteine (g)	0–1
Arginine (g)	8–8.8
Glycine (g)	3.5–4
Histidine (g)	2.3–2.5
Isoleucine (g)	3.5–4.4
Leucine (g)	7.1–7.6
Phenylalanine (g)	5.5–6.6
Tyrosine (g)	3–3.3
Threonine (g)	3.4–3.5
Tryptophan (g)	0–0.9
Valine (g)	3.9–4.6
Alanine (g)	3.7–4.1
Aspartic acid (g)	10–11
Glutamic acid (g)	16–17
Proline (g)	4–4.3
Serine (g)	4.8–5.2

**Table 2:** QTLs generated for *Ascochyta* blight and *Fusarium* wilt resistance.

QTL	Marker	Reference(s)
***Ascochyta blight*** **resistance**		
1	GAA47	[54]
2	TA72, GA2	
ar2	TA72	[55]
	TA146	
I	STMS11, GA2, GAA47, TR20 4	
1	TS12b	[56]
2/3	TA3a/TA3b	
4/5/6	TA30/TA146/TR20	
QTL-2	TA3a	
	TA146	
QTL-2/QTL-3	TA72	
	GA2	
	TA3a/TA3b	
ar1	GA16 2	[57]
ar2a	GA16	
ar2b	TA130, TA72, TS72	
ar1b	TA37, TA200	
ar2a	GA24, GAA47	[58]
ar2b	TA130	
	TA72	
	TS72	
ar19	TR19	
	GA16	
QTL-1	GAA47	[54,56,59]
	TS12b	
	STMS28	
	STMS11	
	GA2	
	TS12b	
	TR20	
QTL-3	TS45	[57,60,61]
	TA3b	
	TA194	
	TS82	
	TR58	
ar1a	GA16	[57,59]
	GA20	
ar1b	TA37	[57]
	TA200	
ar2a	GA16	[59]
	GA24	
	GAA47	
ar1	GA16	
ar1a	GA20, GA16	
qab-4.1, qab-4.2LG7, qab-7.1	qab-4.1: CNC_021163.1.32280291, CNC_021163.1.37933917 qab-4.2: CNC_021163.1.23799836 CNC_021163.1.24184658 qab-7.1: CNC_021166.1.34330294 CNC_021166.1.34330283	[62]
QTL1	Ca_Ce_18445 [Ca_Ce_18577 & Ca_Ce_18594] Ca_Ce_18656	[63]
ar2	SC/OPK13603 4 SC/OPM02935 TA72, TA146	[60,61]
***Fusarium wilt*** **resistance**		
Foc-0/foc-0	TR59	[64]
foc-1	TA59	
	TA96	
	TA27	
foc-2	TA96	
	TA27	
	TR19	
Foc-3/foc-3	TA96	
	TA27	
	TR59	
foc-4	TA59	
	TA96	
	TA27	
	TR19	
	TA194	
Foc-5/foc-5	TA27	
	TA59	
	TA96	
	TA110	
	TA59	
	TA53	
	TA103	
	TS82	
	TR58	
Foc 1 & 3	GA 16	[65]
	TAA60	
	TA194	
	TS82	
	TA110	
	TR19	

**Table 3:** Reports on crop improvement through transformation regimes.

Gene Transferred	Source of Gene	Transformation Method	Explant	Trait Introduced	Expression Level	Reference(s)
**Biotic stress**						
*cryIAc*+ *npt*II+ *CaMV35S*	*Bacillus thuringiensis*	*A. tumefaciens*	Embryo axis	*H. armigera* resistance	Inhibits the development of *Heliothis armigera* larvae	[74]
*cry1Ac* + *npt*II +*CaMV35S*	*Bacillus thuringiensis*	*A. tumefaciens*	Cotyledon nodes	*H. armigera* resistance	Cry1Ac protein showed 80–85% protection with high mortality rate i.e., >80%	[75]
*αAI1*+ *npt*II+ *CaMV3S*	*Phaseolus—vulgaris*	*A. tumefaciens*	Embryogeni c axis	Bruchids resistance	Larval growth reduction	[73]
*cry1Ac* + *npt*II+ *CaMV35S*	*Bacillus thuringiensis*	Particle gun bombardment	Embryonal axis, Epicotyl and stem	Protection from *H. armigera* and *S. litura*	Higher mortality of *Heliothis* *armigera* and *Spodoptera litura* larvae	[76]
*ASAL*+ *npt*II+ *CaMV35S* + *rol*C	*Allium sativum* leaf agglutinin	*A. tumefaciens*	Single cotyledon with half embryo	*Aphis craccivora* resistance	Increase in mortality rate upto 42%	[77]
*CryIAc+ npt*II	*Bacillus thuringiensis*	*A. tumefaciens*	-	*H. armigera* resistance	Mortality of >60% for *H. armigera*	[9]
*cry2Aa*+ *npt*II + *ats*1A	*Bacillus thuringiensis*	*A. tumefaciens*	Cotyledon nodes	*H. armigera* resistance	Showed higher toxicity to the insect.	[69]
*cryIAc+ npt*II*+ uid*A*+ CaMV35S*	*Bacillus thuringiensis*	*A. tumefaciens*	Embryonic axis, epicotyl and stem explants	*H. armigera* resistance	Tolerance to infection by *H. armigera*	[78]
*cry1Ac+ nptII+ uid*A *+ rbcS+ CaMV35S*	*Bacillus thuringiensis*	*A. tumefaciens*	Cotyledon nodes	*H. armigera* resistance	High level protection against pod borer	[79]
*cry1Ab* and *cry1Ac*+ *CaMV35S* or *Pcec*+ *npt*II	*Bacillus thuringiensis*	*A. tumefaciens*	Cotyledon nodes	*H. armigera* resistance	Showed higher mortality of the insect (95%).	[10]
*cry1Ab/Ac+ actin1+ msg*	*Bacillus thuringiensis*	*A. tumefaciens*	Cotyledon nodes	*H. armigera* resistance	Showed higher toxicity to the Pod borer.	[80]
*cryIIAa+ npt*II + *CaMV35S*	*Bacillus thuringiensis*	*A. tumefaciens*	Cotyledon nodes	*H. armigera* resistance	Showed higher toxicity to the insect.	[81]
*cry1Aabc+ npt*II	*Bacillus thuringiensis*	*A. tumefaciens*	Cotyledon nodes	*H. armigera* resistance	Highly effective against pod borer	[72]
*ChTI+ npt*II *+ CaMV35S+* *nos*	*Cocculus hirsutus*	*A. tumefaciens*	Cotyledon nodes	Protection from *H. armigera* and *S. litura*	Showed mortality rate of 60–80%	[70]
**Abiotic stress**						
*P5CSF129A*+ *npt*II + *uid*A+ *CaMV 35S*	*Vigna aconitifolia*	*A. tumefaciens*	Axillary meristem	Drought tolerance	Enhanced proline overcame the adverse effects of drought stress	[82]
*P5CS*+ *hpt* + *CaMV35S*	*Vigna aconitifolia*	*A. tumefaciens*	Cotyledon node	Salt tolerance	Proline overproduction alleviated salt stress	[83]
*AtDREB1A* + *rd29*A promoter	*Arabidopsis thaliana*	*A. tumefaciens*	Axillary meristem	Transpiration efficiency under drought stress	Increased transpiration efficiency	[84]
*PDH45+ hpt+ CaMV 35S*	*Pea DNA * *Helicase 45*	*A. tumefaciens*	Zygotic embryo, decapitated embryo and decapitated embryo with single cotyledon disc	Salt tolerance	Alleviated salt stress	[85]
*miR408* (over expression)	*Arabidopsis thaliana*	Terrestrial plants	Mature embryo	Drought tolerance	Increased drought tolerance	[86]
*AtDREB1a+ rd29a* promoter	*Arabidopsis thaliana*	*A. tumefaciens*	Cotyledon with half embryo axis	Drought tolerance	Enhanced drought tolerance	[87]
*CAMTA* (over expression)	*Gossypium herbaceum*	*A. tumefaciens*	Cotyledon nodes	Salinity and drought stress	Enhanced activities of antioxidant enzymes under drought and salinity	[68]
*CaPDZ1* (Over expression)	*Cicer arietinum*	*A. tumefaciens*	Single cotyledon with embryo	Dehydration tolerance	Conferred dehydration tolerance by improving photosynthesis	[88]
**Nutritional enhancement**						
*SSA*+ *CaMV 35S*+ *uidA+* pea vicilin gene	Sunflower seed albumin gene (*Brassica napus*)	*A. tumefaciens*	Embryo axis	Increased methionine content	Increased methionine content in normal soil state	[69,89]

**Table 4:** Biofortification of chickpea (*Cicer arietinum* L.) through foliar method.

Treatment	Source	Trait Transferred	Expression Level	Reference(s)
Foliar application of Zn	ZnSO_4_.7H_2_O (33% Zn)	Zn biofortification	Increased Zn content in seeds	[102]
Foliar application of Zn	0.1% ZnSO_4_ foliar spray	Efficiency of chickpea	Increased Zn content in seeds	[94]
Foliar application of Se	Sodium selenate and Sodium selenite at four rates (0, 10, 20, 40 g ha^−1^)	Se biofortification	Selenomethionine was found in high concentrations in chickpea grains (>70%).	[103]
Foliar application of Zn-EDTA	Zn-EDTA three sprays (V + F + G)	Zinc Biofortification	Enrichment of seed with Zn	[99]
Foliar application of Zn + urea	ZnSO_4_ @ 0.5% + @ 2% urea	Biofortification of chickpea with Zn and Fe	Enrichment of seed with Zn and Fe	[101]
Foliar application of Zn and Fe	Zn @ 0.5% + Fe @ 0.1%	Biofortification of chickpea with Zn and Fe	Enrichment of chickpeas with Fe and Zn	[104]
Foliar application of ZnO NPs + Fe_2_O_3_ NPs	0.5% ZnO NPs + 0.5% Fe_2_O_3_	Biofortification of chickpea with Zn and Fe	Enrichment of chickpeas with Zn and Fe	[105]

**Table 5:** Crop improvement of chickpea (*Cicer arietinum*L.) by microbial treatment.

Treatment	PGPB	Trait Transferred	Expression Level	Reference(s)
Zn + PGPB	*Enterobacter* sp. MN17	Zn biofortification	Enhanced Zn content in seed	[98,100]
Fe + PGPR (plant growth promoting rhizobacteria)	*Bacillus cereus* UW 85, *Azotobacter vinelandi* MAC 259, *Pseudomonas*, *Bacillus megaterium*, *E. coli*	Fe biofortification	Enhanced Fe content and 81–75% increase in productivity	[106]
*Rhizobium* sps. BHURC01 + PGPR + *Pseudomonas fluorescens*	*Azotobacter chroococcum*, *Bacillus megaterium*	Plant biomass and yield	Inhibited the phytopathogenic fungi leading to suppression of plant disease, Promotion of plant growth and nodule formation.	[107]
Boron coated seed + PGPB	*Bacillus* sp. MN54	Boron efficiency	Increased B content, nodulation and yield	[108]
Zinc-solubilizing bacteria	ZnSB13	Zinc biofortifcation in chickpea	Increased Zn content in seeds	[109]
Zinc-solubilizing bacteria	*B. altitudinis* (BT3 and CT8)	Zinc biofortifcation in chickpea	Improved Zn uptake by 3.9–6.0%.	[8]
Zinc-solubilizing bacteria	*Pseudomonas protegens* (RY2, MF351762)	Zinc biofortifcation in chickpea	Enhanced Zn in soil	[110]

**Table 6:** Biochemical analysis of selected chickpea varieties.

Parameter Tested	Trend Observed
Crude fiber	GPF 2 > PBG 8 > *Cicer judaicum* 1 LWC 185 > BGM 20211 > Pusa 372 > WR 315 > Pusa 391 > ICC 4958 > BGM 10216
Crude protein	BGM 20211 > PBG 8 > Pusa 372 > WR 315 > ICC 4958 > Pusa 391 > BGM 10216 > GPF 2 > *Cicer judaicum* 1 LWC 185
Total ash	PBG 8 > Pusa 372 > GPF 2 > Pusa 391 > BGM 20211 = BGM 10216 > WR 315 > ICC 4958 > *Cicer judaicum* 1 LWC 185
Carbohydrates	GPF 2 > WR 315 > ICC 4958 > BGM 10216 > Pusa 391 > BGM 20211 = Pusa 372 > *Cicer judaicum* 1 LWC 185 > PBG 8
Crude lipid—fat	PBG 8 > Pusa 391 > ICC 4958 > *Cicer judaicum* 1 LWC 185 > Pusa 372 > GPF 2 > WR 315 > BGM 20211 > BGM 10216
Methionine	ICC 4958 > BGM 20211 > Pusa 372 > BGM 10216 > PBG 8 > WR 315 > Pusa 391 > GPF 2 > *Cicer judaicum* 1 LWC 185
Arginine	ICC 4958 > Pusa 372 > BGM 20211 = WR 315 > BGM 10216 > Pusa 391 > PBG 8 > GPF 2 > *Cicer judaicum* 1 LWC 185
Lysine	Pusa 391 > ICC 4958 > Pusa 372 > BGM 20211 > BGM 10216 > WR 315 > PBG 8 > GPF 2 > *Cicer judaicum* 1 LWC 185
Cysteine	BGM 20211 > ICC 4958 > Pusa 372 > BGM 10216 > WR 315 > Pusa 391 > PBG 8 > GPF 2 > *Cicer judaicum* 1 LWC 185
Saturated fatty acids	PBG 8 > Pusa 391 > ICC 4958 > BGM 20211 > Pusa 372 > WR 315 > GPF 2 > BGM 10216 = *Cicer judaicum* 1 LWC 185
Polyunsaturated fatty acids	PBG 8 > GPF 2 > Pusa 391 > ICC 4958 > WR 315 > Pusa 372 > *Cicer judaicum* 1 LWC 185 > BGM 20211 > BGM 10216
Riboflavin	Pusa 391 > BGM 20211 > GPF 2 = ICC 4958 > Pusa 372 > WR 315 > *Cicer judaicum* 1 LWC 185 > PBG 8 > BGM 10216
Niacin	PBG 8 > WR 315 > *Cicer judaicum* 1 LWC 185 = GPF 2 > Pusa 391 > BGM 20211 = Pusa 372 > ICC 4958 > BGM 10216
Thiamin	BGM 10216 > Pusa 372 > PBG 8 > WR 315 > ICC 4958 > Pusa 391 > GPF 2 > BGM 20211 > *Cicer judaicum* 1 LWC 185
Folate	WR 315 > BGM 20211 > BGM 10216 > Pusa 391 > Pusa 372 = ICC 4958 > PBG 8 > GPF 2 > *Cicer judaicum* 1 LWC 185
B—Carotene (vitamin A)	BGM 20211 > ICC 4958 = PBG 8 > Pusa 372 > Pusa 391 = *Cicer judaicum* 1 LWC 185 > WR 315 > BGM 10216 > GPF 2
Phenolics	PBG 8 > WR 315 > Pusa 391 > GPF 2 > BGM 10216 > *Cicer judaicum* 1 LWC 185 > ICC 4958 > BGM 20211 > Pusa 372
Flavanoids	GPF 2 = BGM 10216 > Pusa 391 > ICC 4958 = PBG 8 > WR 315 > Pusa 372 = *Cicer judaicum* 1 LWC 185 > BGM 20211
Omega 6: Omega 3	Pusa 391 > BGM 20211 > BGM 10216 > ICC 4958 = WR 315 = PBG 8 > GPF 2 > Pusa 372 > *Cicer judaicum* 1 LWC 185
Antioxidant activity	BGM 10216 = BGM 20211 > PBG 8 > GPF 2 > WR 315 > *Cicer judaicum* 1 LWC 185 > ICC 4958 > Pusa 372 > Pusa 391
Lectin and hemaglutination assay	PBG 8 > GPF 2 > WR 315 > Pusa 391 > ICC 4958 > *Cicer judaicum* 1 LWC 185 > BGM 20211 > BGM 10216 > Pusa 372
Uric acid	BGM 20211 > ICC 4958 > BGM 10216 = PBG 8 > GPF 2 > Pusa 372 > Pusa 391 > WR 315 > *Cicer judaicum* 1 LWC 185
Energy value	ICC 4958 > Pusa 391 = GPF 2 > WR 315 > BGM 20211 > Pusa 372 > BGM 10216 > PBG 8 > *Cicer judaicum* 1 LWC 185
Protein bioavailability	BGM 20211 > BGM 10216 > Pusa 391 > PBG 8 > WR 315 > GPF 2 > ICC 4958 > *Cicer judaicum* 1 LWC 185 > Pusa 372
Phytic acid	Pusa 372 = PBG 8 > BGM 20211 = WR 315 > *Cicer judaicum* 1 LWC 185 = ICC 4958 > GPF 2 > BGM 10216 > Pusa 391

## Data Availability

Not applicable.

## References

[1] Gaur P.M., Tripathi S., Gowda C.L., Ranga Rao G., Sharma H., Pande S., Sharma M. (2010). Chickpea Seed Production Manual.

[2] Rasheed A., Gill R.A., Hassan M.U., Mahmood A., Qari S., Zaman Q.U., Ilyas M., Aamer M., Batool M., Li H. (2021). A critical review: Recent advancements in the use of CRISPR/Cas9 technology to enhance crops and alleviate global food crises. Curr. Issues Mol. Biol..

[3] Grasso N., Lynch N.L., Arendt E.K., O’Mahony J.A. (2022). Chickpea protein ingredients: A review of composition, functionality, and applications. Compr. Rev. Food Sci. Food Saf..

[4] Ladizinsky G., Adler A. (1976). The origin of chickpea *Cicer arietinum* L. Euphytica.

[5] Acharjee S., Sarmah B.K. (2013). Biotechnologically generating ‘super chickpea’ for food and nutritional security. Plant Sci..

[6] Roorkiwal M., Bharadwaj C., Barmukh R., Dixit G.P., Thudi M., Gaur P.M., Chaturvedi S.K., Fikre A., Hamwieh A., Kumar S. (2020). Integrating genomics for chickpea improvement: Achievements and opportunities. Theor. Appl. Genet..

[7] Benkadri S., Salvador A., Zidoune M.N., Sanz T. (2018). Gluten-free biscuits based on composite rice—Chickpea flour and xanthan gum. Food Sci. Technol. Int..

[8] Kushwaha P., Srivastava R., Pandiyan K., Singh A., Chakdar H., Kashyap P.L., Bhardwaj A.K., Murugan K., Karthikeyan N., Bagul S.Y. (2021). Enhancement in plant growth and zinc biofortification of chickpea (*Cicer arietinum* L.) by *Bacillus altitudinis*. J. Soil Sci. Plant Nutr..

[9] Biradar S.S., Sridevi O., Salimath P. (2009). Genetic enhancement of chickpea for pod borer resistance through expression of *CryIAc* protein. Karnataka J. Agric. Sci..

[10] Mehrotra M., Singh A.K., Sanyal I., Altosaar I., Amla D. (2011). Pyramiding of modified *cry1Ab* and *cry1Ac* genes of *Bacillus thuringiensis* in transgenic chickpea (*Cicer arietinum* L.) for improved resistance to pod borer insect *Helicoverpa armigera*. Euphytica.

[11] Summerfield R., Roberts E. (1988). Photo-thermal regulation of flowering in pea, lentil, faba bean and chickpea. World Crops: Cool Season Food Legumes.

[12] Yadav S., Longnecker N., Dusunceli F., Bejiga G., Yadav M., Rizvi A., Manohar M., Reddy A., Xaxiao Z., Chen W. (2007). Uses, consumption and utilization. Chickpea Breeding and Management.

[13] Jain M., Misra G., Patel R.K., Priya P., Jhanwar S., Khan A.W., Shah N., Singh V.K., Garg R., Jeena G. (2013). A draft genome sequence of the pulse crop chickpea (*Cicer arietinum* L.). Plant J..

[14] López-Bellido F.J., López-Bellido R.J., Khalil S.K., López-Bellido L. (2008). Effect of planting date on winter kabuli chickpea growth and yield under rainfed Mediterranean conditions. Agron. J..

[15] De Santis M.A., Rinaldi M., Menga V., Codianni P., Giuzio L., Fares C., Flagella Z. (2021). Influence of organic and conventional farming on grain yield and protein composition of chickpea genotypes. Agronomy.

[16] Kaur M., Singh N. (2005). Studies on functional, thermal and pasting properties of flours from different chickpea (*Cicer arietinum* L.) cultivars. Food Chem..

[17] Singh M., Bisht I.S., Dutta M. (2014). Broadening the Genetic Base of Grain Legumes.

[18] Heuzé V., Tran G., Boudon A., Bastianelli D., Lebas F. (2015). Chickpea (Cicer Arietinum). https://www.feedipedia.org/node/319.

[19] Jukanti A.K., Gaur P.M., Gowda C., Chibbar R.N. (2012). Nutritional quality and health benefits of chickpea (*Cicer arietinum* L.): A review. Br. J. Nutr..

[20] Keyimu X.G., Bozlar M.A., Wulamujiang A. (2020). Pharmacology properties of *Cicer arietinum* L. Int. J. ChemTech Res..

[21] Faridy J.-C.M., Stephanie C.-G.M., Gabriela M.-M.O., Cristian J.-M. (2020). Biological activities of chickpea in human health (*Cicer arietinum* L.). A review. Plant Foods Hum. Nutr..

[22] Guillon F., Champ M.-J. (2002). Carbohydrate fractions of legumes: Uses in human nutrition and potential for health. Br. J. Nutr..

[23] Barmukh R., Soren K.R., Madugula P., Gangwar P., Shanmugavadivel P., Bharadwaj C., Konda A.K., Chaturvedi S.K., Bhandari A., Rajain K. (2021). Construction of a high-density genetic map and QTL analysis for yield, yield components and agronomic traits in chickpea (*Cicer arietinum* L.). PLoS ONE.

[24] Gaur P.M., Jukanti A.K., Varshney R.K. (2012). Impact of genomic technologies on chickpea breeding strategies. Agronomy.

[25] Jha U.C., Nayyar H., Palakurthi R., Jha R., Valluri V., Bajaj P., Chitikineni A., Singh N.P., Varshney R.K., Thudi M. (2021). Major QTLs and potential candidate genes for heat stress tolerance identified in chickpea (*Cicer arietinum* L.). Front. Plant Sci..

[26] Varshney R.K., Roorkiwal M., Sun S., Bajaj P., Chitikineni A., Thudi M., Singh N.P., Du X., Upadhyaya H.D., Khan A.W. (2021). A chickpea genetic variation map based on the sequencing of 3366 genomes. Nature.

[27] Upadhyaya H.D., Thudi M., Dronavalli N., Gujaria N., Singh S., Sharma S., Varshney R.K. (2011). Genomic tools and germplasm diversity for chickpea improvement. Plant Genet. Resour..

[28] Bharadwaj C., Sachdeva S., Singh R.K., Patil B., Roorkiwal M., Chaturvedi S., Varshney R. (2018). Chickpea Genomics. Biotechnologies of Crop Improvement.

[29] Nguyen D.T., Hayes J.E., Atieno J., Li Y., Baumann U., Pattison A., Bramley H., Hobson K., Roorkiwal M., Varshney R.K. (2022). The genetics of vigour-related traits in chickpea (*Cicer arietinum* L.): Insights from genomic data. Theor. Appl. Genet..

[30] Gaur P.M., Thudi M., Samineni S., Varshney R.K. (2014). Advances in chickpea genomics. Legumes in the Omic Era.

[31] Thudi M., Bohra A., Nayak S., Varghese N., Shah T., Penmetsa R., Thirunavukkarasu N., Gudipati S., Gaur P., Kulwal P. (2011). Novel SSR Markers from BAC-End Sequences, DArT Arrays and a Comprehensive Genetic Map with 1291 Marker Loci for Chickpea (*Cicer arietinum* L.). PLoS ONE.

[32] Madrid E., Seoane P., Claros M., Barro F., Rubio J., Gil J., Millán T. (2014). Genetic and physical mapping of the QTLAR3 controlling blight resistance in chickpea (*Cicer arietinum* L). Euphytica.

[33] Jha U.C. (2018). Current advances in chickpea genomics: Applications and future perspectives. Plant Cell Rep..

[34] Lichtenzveig J., Scheuring C., Dodge J., Abbo S., Zhang H.-B. (2005). Construction of BAC and BIBAC libraries and their applications for generation of SSR markers for genome analysis of chickpea, *Cicer arietinum* L. Theor. Appl. Genet..

[35] Coram T.E., Pang E.C. (2005). Isolation and analysis of candidate Ascochyta blight defence genes in chickpea. Part I. Generation and analysis of an expressed sequence tag (EST) library. Physiol. Mol. Plant Pathol..

[36] Hiremath P.J., Farmer A., Cannon S.B., Woodward J., Kudapa H., Tuteja R., Kumar A., BhanuPrakash A., Mulaosmanovic B., Gujaria N. (2011). Large-scale transcriptome analysis in chickpea (*Cicer arietinum* L.), an orphan legume crop of the semi-arid tropics of Asia and Africa. Plant Biotechnol. J..

[37] Nayak S.N., Zhu H., Varghese N., Datta S., Choi H.-K., Horres R., Jüngling R., Singh J., Kavi Kishor P., Sivaramakrishnan S. (2010). Integration of novel SSR and gene-based SNP marker loci in the chickpea genetic map and establishment of new anchor points with *Medicago truncatula* genome. Theor. Appl. Genet..

[38] Varshney R.K., Song C., Saxena R.K., Azam S., Yu S., Sharpe A.G., Cannon S., Baek J., Rosen B.D., Tar’an B. (2013). Draft genome sequence of chickpea (*Cicer arietinum*) provides a resource for trait improvement. Nat. Biotechnol..

[39] Jaccoud D., Peng K., Feinstein D., Kilian A. (2001). Diversity arrays: A solid state technology for sequence information independent genotyping. Nucleic Acids Res..

[40] Katna G., Nitesh S., Sharma K.D. (2020). Conventional cytogenetic manipulations. Chickpea: Crop Wild Relatives for Enhancing Genetic Gains.

[41] Gujaria N., Kumar A., Dauthal P., Dubey A., Hiremath P., Bhanu Prakash A., Farmer A., Bhide M., Shah T., Gaur P.M. (2011). Development and use of genic molecular markers (GMMs) for construction of a transcript map of chickpea (*Cicer arietinum* L.). Theor. Appl. Genet..

[42] Korte A., Farlow A. (2013). The advantages and limitations of trait analysis with GWAS: A review. Plant Methods.

[43] Srungarapu R., Mahendrakar M.D., Mohammad L.A., Chand U., Jagarlamudi V.R., Kondamudi K.P., Kudapa H., Samineni S. (2022). Genome-Wide Association Analysis Reveals Trait-Linked Markers for Grain Nutrient and Agronomic Traits in Diverse Set of Chickpea Germplasm. Cells.

[44] Ahmed S.M., Alsamman A.M., Jighly A., Mubarak M.H., Al-Shamaa K., Istanbuli T., Momtaz O.A., El Allali A., Hamwieh A. (2021). Genome-wide association analysis of chickpea germplasms differing for salinity tolerance based on DArTseq markers. PLoS ONE.

[45] Gaur P., Slinkard A. (1990). Genetic control and linkage relations of additional isozyme markers in chick-pea. Theor. Appl. Genet..

[46] Winter P., Benko-Iseppon A.-M., Hüttel B., Ratnaparkhe M., Tullu A., Sonnante G., Pfaff T., Tekeoglu M., Santra D., Sant V. (2000). A linkage map of the chickpea (*Cicer arietinum* L.) genome based on recombinant inbred lines from a *C. arietinum* × *C. reticulatum* cross: Localization of resistance genes for fusarium wilt races 4 and 5. Theor. Appl. Genet..

[47] Simon C., Muehibauer F. (1997). Construction of a chickpea linkage map and its comparison with maps of pea and lentil. J. Hered..

[48] Anuradha C., Gaur P.M., Pande S., Gali K.K., Ganesh M., Kumar J., Varshney R.K. (2011). Mapping QTL for resistance to botrytis grey mould in chickpea. Euphytica.

[49] Garg V., Kaushik P. (2006). Influence of short-term irrigation of textile mill wastewater on the growth of chickpea cultivars. Chem. Ecol..

[50] Millan T., Winter P., Jüngling R., Gil J., Rubio J., Cho S., Cobos M., Iruela M., Rajesh P., Tekeoglu M. (2010). A consensus genetic map of chickpea (*Cicer arietinum* L.) based on 10 mapping populations. Euphytica.

[51] Rajesh P., Coyne C., Meksem K., Sharma K., Gupta V., Muehlbauer F. (2004). Construction of a *Hind*III bacterial artificial chromosome library and its use in identification of clones associated with disease resistance in chickpea. Theor. Appl. Genet..

[52] Zhang X., Scheuring C.F., Zhang M., Dong J.J., Zhang Y., Huang J.J., Lee M.-K., Abbo S., Sherman A., Shtienberg D. (2010). A BAC/BIBAC-based physical map of chickpea, *Cicer arietinum* L. BMC Genom..

[53] Mazumdar D., Saha S.P., Ghosh S. (2020). Isolation, screening and application of a potent PGPR for enhancing growth of Chickpea as affected by nitrogen level. Int. J. Veg. Sci..

[54] Tekeoglu M., Santra D.K., Kaiser W.J., Muehlbauer F.J. (2000). Ascochyta blight resistance inheritance in three chickpea recombinant inbred line populations. Crop Sci..

[55] Rakshit S., Winter P., Tekeoglu M., Juarez Muñoz J., Pfaff T., Benko-Iseppon A.-M., Muehlbauer F., Kahl G. (2003). DAF marker tightly linked to a major locus for Ascochyta blight resistance in chickpea (*Cicer arietinum* L.). Euphytica.

[56] Flandez-Galvez H., Ford R., Pang E., Taylor P. (2003). An intraspecific linkage map of the chickpea (*Cicer arietinum* L.) genome based on sequence tagged microsatellite site and resistance gene analog markers. Theor. Appl. Genet..

[57] Udupa S.M., Baum M. (2003). Genetic dissection of pathotype-specific resistance to ascochyta blight disease in chickpea (*Cicer arietinum* L.) using microsatellite markers. Theor. Appl. Genet..

[58] Collard B.C., Pang E.C., Ades P.K., Taylor P.W. (2003). Preliminary investigation of QTLs associated with seedling resistance to ascochyta blight from *Cicer echinospermum*, a wild relative of chickpea. Theor. Appl. Genet..

[59] Cho S., Chen W., Muehlbauer F.J. (2004). Pathotype-specific genetic factors in chickpea (*Cicer arietinum* L.) for quantitative resistance to ascochyta blight. Theor. Appl. Genet..

[60] Millan T., Rubio J., Iruela M., Daly K., Cubero J., Gil J. (2003). Markers associated with Ascochyta blight resistance in chickpea and their potential in marker-assisted selection. Field Crops Res..

[61] Iruela M., Rubio J., Cubero J., Gil J., Millan T. (2002). Phylogenetic analysis in the genus *Cicer* and cultivated chickpea using RAPD and ISSR markers. Theor. Appl. Genet..

[62] Kushwah A., Bhatia D., Singh I., Thudi M., Singh G., Bindra S., Vij S., Gill B., Bharadwaj C., Singh S. (2021). Identification of stable heat tolerance QTLs using inter-specific recombinant inbred line population derived from GPF 2 and ILWC 292. PLoS ONE.

[63] Sudheesh S., Kahrood H.V., Braich S., Dron N., Hobson K., Cogan N.O., Kaur S. (2021). Application of genomics approaches for the improvement in ascochyta blight resistance in chickpea. Agronomy.

[64] Sharma K.D., Muehlbauer F.J. (2007). Fusarium wilt of chickpea: Physiological specialization, genetics of resistance and resistance gene tagging. Euphytica.

[65] Varshney R.K., Thudi M., Nayak S.N., Gaur P.M., Kashiwagi J., Krishnamurthy L., Jaganathan D., Koppolu J., Bohra A., Tripathi S. (2014). Genetic dissection of drought tolerance in chickpea (*Cicer arietinum* L.). Theor. Appl. Genet..

[66] Razzaq M.K., Akhter M., Ahmad R.M., Cheema K.L., Hina A., Karikari B., Raza G., Xing G., Gai J., Khurshid M. (2022). CRISPR-Cas9 based stress tolerance: New hope for abiotic stress tolerance in chickpea (*Cicer arietinum*). Mol. Biol. Rep..

[67] Amiri S.R., Deihimfard R., Eyni-Nargeseh H. (2020). Toward dormant seeding of rainfed chickpea as an adaptation strategy to sustain productivity in response to changing climate. Field Crops Res..

[68] Kumar A., Kumar V., Dubey A.K., Narayan S., Sawant S.V., Pande V., Shirke P.A., Sanyal I. (2022). CAMTA transcription factor enhances salinity and drought tolerance in chickpea (*Cicer arietinum* L.). Plant Cell Tissue Organ Cult. (PCTOC).

[69] Acharjee S., Sarmah B.K., Kumar P.A., Olsen K., Mahon R., Moar W.J., Moore A., Higgins T. (2010). Transgenic chickpeas (*Cicer arietinum* L.) expressing a sequence-modified *cry2Aa* gene. Plant Sci..

[70] Pandey A., Yadav R., Kumar S., Kumar A., Shukla P., Yadav A., Sanyal I. (2021). Expression of the entomotoxic *Cocculus hirsutus* trypsin inhibitor (ChTI) gene in transgenic chickpea enhances its underlying resistance against the infestation of *Helicoverpa armigera* and *Spodoptera litura*. Plant Cell Tissue Organ Cult. (PCTOC).

[71] Kaiser W., Schaad N., Mink G., Hampton R. (1988). Workshop: Seed pathogens of food legumes. World Crops: Cool Season Food Legumes.

[72] Das A., Datta S., Thakur S., Shukla A., Ansari J., Sujayanand G., Chaturvedi S.K., Kumar P., Singh N. (2017). Expression of a chimeric gene encoding insecticidal crystal protein Cry1Aabc of *Bacillus thuringiensis* in chickpea (*Cicer arietinum* L.) confers resistance to gram pod borer (*Helicoverpa armigera* Hubner.). Front. Plant Sci..

[73] Ignacimuthu S., Prakash S. (2006). *Agrobacterium*-mediated transformation of chickpea with α-amylase inhibitor gene for insect resistance. J. Biosci..

[74] Kar S., Basu D., Das S., Ramkrishnan N.A., Mukherjee P., Nayak P., Sen S.K. (1997). Expression of *cryIA (c)* gene of *Bacillus thuringiensis* in transgenic chickpea plants inhibits development of pod-borer (*Heliothis armigera*) larvae. Transgenic Res..

[75] Sanyal I., Singh A.K., Kaushik M., Amla D.V. (2005). *Agrobacterium*-mediated transformation of chickpea (*Cicer arietinum* L.) with *Bacillus thuringiensis cry1Ac* gene for resistance against pod borer insect *Helicoverpa armigera*. Plant Sci..

[76] Indurker S., Misra H.S., Eapen S. (2007). Genetic transformation of chickpea (*Cicer arietinum* L.) with insecticidal crystal protein gene using particle gun bombardment. Plant Cell Rep..

[77] Chakraborti D., Sarkar A., Mondal H.A., Das S. (2009). Tissue specific expression of potent insecticidal, *Allium sativum* leaf agglutinin (ASAL) in important pulse crop, chickpea (*Cicer arietinum* L.) to resist the phloem feeding *Aphis craccivora*. Transgenic Res..

[78] Indurker S., Misra H.S., Eapen S. (2010). *Agrobacterium*-mediated transformation in chickpea (*Cicer arietinum* L.) with an insecticidal protein gene: Optimization of different factors. Physiol. Mol. Biol. Plants.

[79] Chakraborty J., Sen S., Ghosh P., Sengupta A., Basu D., Das S. (2016). Homologous promoter derived constitutive and chloroplast targeted expression of synthetic *cry1Ac* in transgenic chickpea confers resistance against *Helicoverpa armigera*. Plant Cell Tissue Organ Cult. (PCTOC).

[80] Ganguly M., Molla K.A., Karmakar S., Datta K., Datta S.K. (2014). Development of pod borer-resistant transgenic chickpea using a pod-specific and a constitutive promoter-driven fused *cry1Ab/Ac* gene. Theor. Appl. Genet..

[81] Sawardekar S., Katageri I., Salimath P., Kumar P., Kelkar V. (2017). Standardization of in-vitro genetic transformation technique in chickpea (*Cicer arietinum* L.) for pod-borer resistance. Adv. Agric. Res. Technol. J.

[82] Bhatnagar-Mathur P., Vadez V., Jyostna Devi M., Lavanya M., Vani G., Sharma K.K. (2009). Genetic engineering of chickpea (*Cicer arietinum* L.) with the *P5CSF129A* gene for osmoregulation with implications on drought tolerance. Mol. Breed..

[83] Kiran Kumar Ghanti S., Sujata K., Kumar V., Nataraja Karba N., Srinath Rao M., Kavi Kishor P. (2011). Heterologous expression of *P5CS* gene in chickpea enhances salt tolerance without affecting yield. Biol. Plant..

[84] Anbazhagan K., Bhatnagar-Mathur P., Sharma K.K., Baddam R., Kishor P.K., Vadez V. (2014). Changes in timing of water uptake and phenology favours yield gain in terminal water stressed chickpea AtDREB1A transgenics. Funct. Plant Biol..

[85] Mubina N., Hoque M., Sarker R. (2018). In vitro Regeneration and Over Expression of Pea DNA Helicase 45 (PDH45) Gene into the Local Cultivars of Chickpea (*Cicer arietinum* L.) through *Agrobacterium*-mediated Genetic Transformation. Plant Tissue Cult. Biotechnol..

[86] Hajyzadeh M., Turktas M., Khawar K.M., Unver T. (2015). miR408 overexpression causes increased drought tolerance in chickpea. Gene.

[87] Das A., Basu P.S., Kumar M., Ansari J., Shukla A., Thakur S., Singh P., Datta S., Chaturvedi S.K., Sheshshayee M. (2021). Transgenic chickpea (*Cicer arietinum* L.) harbouring AtDREB1a are physiologically better adapted to water deficit. BMC Plant Biol..

[88] Lande N.V., Barua P., Gayen D., Wardhan V., Jeevaraj T., Kumar S., Chakraborty S., Chakraborty N. (2022). Dehydration-responsive chickpea chloroplast protein, CaPDZ1, confers dehydration tolerance by improving photosynthesis. Physiol. Plant..

[89] Tabe L., Wirtz M., Molvig L., Droux M., Hell R. (2010). Overexpression of serine acetlytransferase produced large increases in O-acetylserine and free cysteine in developing seeds of a grain legume. J. Exp. Bot..

[90] Romeis J., Sharma H.C., Sharma K.K., Das S., Sarmah B.K. (2004). The potential of transgenic chickpeas for pest control and possible effects on non-target arthropods. Crop Prot..

[91] Lingappa S., Hegde R. (2001). Exploitation of biocontrol potential in the management of insect pests of pulse crops. Biocontrol Potential and Its Exploitation in Sustainable Agriculture.

[92] Lawo N., Mahon R., Milner R., Sarmah B., Higgins T., Romeis J. (2008). Effectiveness of *Bacillus thuringiensis*-transgenic chickpeas and the entomopathogenic fungus *Metarhizium anisopliae* in controlling *Helicoverpa armigera* (Lepidoptera: Noctuidae). Appl. Environ. Microbiol..

[93] Anbazhagan K., Bhatnagar-Mathur P., Vadez V., Dumbala S.R., Kishor P., Sharma K.K. (2015). DREB1A overexpression in transgenic chickpea alters key traits influencing plant water budget across water regimes. Plant Cell Rep..

[94] Pathak G.C., Gupta B., Pandey N. (2012). Improving reproductive efficiency of chickpea by foliar application of zinc. Braz. J. Plant Physiol..

[95] Laus M.F., Vales L.D.M.F., Costa T.M.B., Almeida S.S. (2011). Early postnatal protein-calorie malnutrition and cognition: A review of human and animal studies. Int. J. Environ. Res. Public Health.

[96] Cynober L., Bier D.M., Stover P., Kadowaki M., Morris S.M., Elango R., Smriga M. (2020). Proposals for upper limits of safe intake for methionine, histidine, and lysine in healthy humans. J. Nutr..

[97] Pellegrino E., Bedini S. (2014). Enhancing ecosystem services in sustainable agriculture: Biofertilization and biofortification of chickpea (*Cicer arietinum* L.) by arbuscular mycorrhizal fungi. Soil Biol. Biochem..

[98] Ullah A., Farooq M., Hussain M. (2020). Improving the productivity, profitability and grain quality of kabuli chickpea with co-application of zinc and endophyte bacteria *Enterobacter* sp. MN17. Arch. Agron. Soil Sci..

[99] Shivay Y.S., Prasad R., Pal M. (2015). Effects of source and method of zinc application on yield, zinc biofortification of grain, and Zn uptake and use efficiency in chickpea (*Cicer arietinum* L.). Commun. Soil Sci. Plant Anal..

[100] Ullah A., Farooq M., Nadeem F., Rehman A., Hussain M., Nawaz A., Naveed M. (2020). Zinc application in combination with zinc solubilizing Enterobacter sp. MN17 improved productivity, profitability, zinc efficiency, and quality of desi chickpea. J. Soil Sci. Plant Nutr..

[101] Pal V., Singh G., Dhaliwal S. (2019). Agronomic biofortification of chickpea with zinc and iron through application of zinc and urea. Commun. Soil Sci. Plant Anal..

[102] Ullah A., Farooq M., Nadeem F., Rehman A., Nawaz A., Naveed M., Wakeel A., Hussain M. (2020). Zinc seed treatments improve productivity, quality and grain biofortification of desi and kabuli chickpea (*Cicer arietinum*). Crop Pasture Sci..

[103] Poblaciones M.J., Rodrigo S., Santamaria O., Chen Y., McGrath S.P. (2014). Selenium accumulation and speciation in biofortified chickpea (*Cicer arietinum* L.) under Mediterranean conditions. J. Sci. Food Agric..

[104] Rathod S., Channakeshava S., Basavaraja B., Shashidhara K. (2020). Effect of soil and foliar application of zinc and Boron on growth, yield and micro nutrient uptake of Chickpea. J. Pharmacogn. Phytochem..

[105] Dhaliwal S.S., Sharma V., Shukla A.K., Verma V., Behera S.K., Singh P., Alotaibi S.S., Gaber A., Hossain A. (2021). Comparative efficiency of mineral, chelated and nano forms of zinc and iron for improvement of zinc and iron in chickpea (*Cicer arietinum* L.) through biofortification. Agronomy.

[106] Saira K., Asghar H.N., Akhtar M.J., Ana A., Zahir Z.A. (2015). Biofortification of iron in chickpea by plant growth promoting rhizobacteria. Pak. J. Bot..

[107] Verma J., Yadav J., Tiwari K.N. (2010). Application of *Rhizobium* sp. BHURC01 and plant growth promoting rhizobacteria on nodulation, plant biomass and yields of chickpea (*Cicer arietinum* L.). Int. J. Agric. Res..

[108] Mehboob N., Yasir T.A., Ul-Allah S., Nawaz A., Ahmad N., Hussain M. (2022). Interactive Effect of Boron Application Methods and Boron-Tolerant Bacteria (*Bacillus* sp. MN54) Improves Nodulation, Grain Yield, Profitability and Biofortification of kabuli Chickpea Grown Under Irrigated and Rainfed Conditions. J. Soil Sci. Plant Nutr..

[109] Batool S., Asghar H.N., Shehzad M.A., Yasin S., Sohaib M., Nawaz F., Akhtar G., Mubeen K., Zahir Z.A., Uzair M. (2021). Zinc-solubilizing bacteria-mediated enzymatic and physiological regulations confer zinc biofortification in chickpea (*Cicer arietinum* L.). J. Soil Sci. Plant Nutr..

[110] Yasmin R., Hussain S., Rasool M.H., Siddique M.H., Muzammil S. (2021). Isolation, characterization of Zn solubilizing bacterium (*Pseudomonas protegens* RY2) and its contribution in growth of chickpea (*Cicer arietinum* L) as deciphered by improved growth parameters and Zn content. Dose Response.

[111] Hussain M., Mehboob N., Naveed M., Shehzadi K., Yasir T.A. (2020). Optimizing boron seed coating level and boron-tolerant bacteria for improving yield and biofortification of chickpea. J. Soil Sci. Plant Nutr..

[112] Yadav R., Mehrotra M., Singh A.K., Niranjan A., Singh R., Sanyal I., Lehri A., Pande V., Amla D. (2017). Improvement in Agrobacterium-mediated transformation of chickpea (*Cicer arietinum* L.) by the inhibition of polyphenolics released during wounding of cotyledonary node explants. Protoplasma.

